# Investigation of the Anticancer Effect of *α*-Aminophosphonates and Arylidine Derivatives of 3-Acetyl-1-aminoquinolin-2(*1H*)-one on the DMBA Model of Breast Cancer in Albino Rats with In Silico Prediction of Their Thymidylate Synthase Inhibitory Effect

**DOI:** 10.3390/molecules27030756

**Published:** 2022-01-24

**Authors:** Mohamed A. Nassan, Adil Aldhahrani, Hamada H. Amer, Ahmed Elhenawy, Ayman A. Swelum, Omar M. Ali, Yasser H. Zaki

**Affiliations:** 1 Department of Clinical Laboratory Sciences, Turabah University College, Taif University, P.O. Box 11099, Taif 21944, Saudi Arabia; m.nassan@tu.edu.sa (M.A.N.); a.ahdhahrani@tu.edu.sa (A.A.); 2 Department of Chemistry, Turabah University College, Taif University, P.O. Box 11099, Taif 21944, Saudi Arabia; om.ali@tu.edu.sa; 3 Department of Chemistry, Faculty of Science, Al-Azhar University, Cairo 11884, Egypt; ahmed.elhenawy@azhar.edu.eg; 4 Department of Theriogenology, Faculty of Veterinary Medicine, Zagazig University, Zagazig 44519, Egypt; aymanswelum@zu.edu.eg; 5 Department of Animal Production, College of Food and Agriculture Sciences, King Saud University, P.O. Box 2460, Riyadh 11451, Saudi Arabia; 6 Department of Chemistry, Faculty of Science, Beni-Suef University, Beni Suef 62514, Egypt; 7 Department of Chemistry, Faculty of Science and Humanity Studies at Al-Quwayiyah, Shaqra University, Al-Quwayiyah 11961, Saudi Arabia

**Keywords:** *α*-aminophosphonates, 3-acetyl-1-aminoquinolin-2(*1H*)-one, breast cancer, DMBA, thymidylate synthase

## Abstract

Breast cancer is a major cause of death in women worldwide. In this study, 60 female rats were classified into 6 groups; negative control, *α*-aminophosphonates, arylidine derivatives of 3-acetyl-1-aminoquinolin-2(*1H*)-one, DMBA, DMBA & *α*-aminophosphonates, and DMBA & arylidine derivatives of 3-acetyl-1-aminoquinolin-2(*1H*)-one. New *α*-aminophosphonates and arylidine derivatives of 3-acetyl-1-aminoquinolin-2(*1H*)-one were synthesized and elucidated by different spectroscopic and elemental analysis. Histopathological examination showed marked proliferation of cancer cells in the DMBA group. Treatment with *α*-aminophosphonates mainly decreased tumor mass. Bcl2 expression increased in DMBA-administered rats and then declined in the treated groups, mostly with α-aminophosphonates. The level of CA15-3 markedly declined in DMBA groups treated with α-aminophosphonates and arylidine derivatives of 3-acetyl-1-aminoquinolin-2(*1H*)-one. Gene expression of GST-P, PCNA, PDK, and PIK3CA decreased in the DMBA group treated with α-aminophosphonates and arylidine derivatives of 3-acetyl-1-aminoquinolin-2(*1H*)-one, whereas PIK3R1 and BAX increased in the DMBA group treated with α-aminophosphonates and arylidine derivatives of 3-acetyl-1-aminoquinolin-2(*1H*)-one. The molecular docking postulated that the investigated compounds can inhibt the Thymidylate synthase TM due to high hydrophobicity charachter.

## 1. Introduction

There are lots of compounds which have therapeutic effects, such as *α*-Aminophosphonates, which have different biological activities such as antibacterial [1], anti-alzheimer disease [2], anti-tumor [3], and anticancer activity [4]. A new compound of phosphonates has been designed, synthesized and tested, diethyl 3-nonyl-5-oxo-3,5,6,6 a-tetrahydro-*1H*-cyclopenta [*c*]furan-4-ylphosphonate (P-5). It has been tested in-vitro for its anti-inflammatory effects and in-vivo for its ability to improve colitis [5]. A series of biphenyl (4′-(arylidiazinyl) diphenyl-4-ylamino) (pyridine-3-yl) methylphosphonates were also synthesized and then their antimicrobial activity was verified. Some compounds showed high activity against *B. subtilis*, *S. aureus*, *Escherichia coli*, *C. albicans,* and *S. cerevisiae* at low concentrations [6]. Some *α*-aminophosphonate derivatives containing a fraction of pyrazole have been prepared and evaluated for COX-2 inhibition and anti-cancer activity. In vitro tests showed excellent COX-2 inhibitory activity and antiproliferative activity against MCF-7 cells, which provided some new ideas in designing therapeutic drugs for COX-2 inhibitors with selective and anti-tumor activity [7].

Other derivatives of *N*-substituted pyrazole from *α*-aminophosphonates was designed, synthesized and evaluated for its anticholinesterase activity. Swine pancreatic lipase (PPL) has been used as an organic transformation promoter. Some synthesized compounds have been shown to be more effective than the standard drugs tacrine, rivastigmine, and galantamine for inhibiting anticholinesterase [2]. New organic compounds have been synthesized from the acylthiourea derivatives containing the pyrazole ring. The synthesized compounds were evaluated as antihistaminics. Cell culture studies have shown significant toxicity to the compounds on cell lines [8]. So our study aims to study the effect of *α*-aminophosphonates and arylidine derivatives of 3-acetyl-1-aminoquinolin-2(*1H*)-one on DMBA model of breast cancer in albino rats with insilico prediction of their thymidylate synthase inhibitory effect.

## 2. Materials and Methods

### 2.1. Chemistry

When preparing new compounds, it is important to measure their melting points by means of a Kofler block device that is not corrected. The proton and carbon NMR spectra were determined by a Varian Gemini 200 NMR spectrometer at 500 MHz with TMS as a reference at King Saud University, Saudi Arabia. The finishing of the reactions was monitored by *TLC* using aluminum silica gel plates at 60 F 245.

**Preparation of dibenzalacetone 3 [9]:**

A methanolic solution was made by dissolving 15 mmol of dry acetone and 30 mmol of methanol. After that, an ice bath was prepared, and the round flask containing the mixture was placed, and then stirred for 3 h while slowly dropping 11% sodium hydroxide solution. The resulting mixture is left to stand for two hours. The resulting yellow crystals are then filtered and recrystallized from methanol to give dibenzalacetone. Yield: 90%, mp. 112 °C.

**Preparation of 5-phenyl-3-[2-phenylvinyl]-4,5-dihydro-*1H*-pyrazole-1-carbaldehyde 4 [9]:**

The ethanolic solution containing 10 mmol of hydrazine hydrate in 5 mL of ethanol was dropped into the mixture consisting of 5 mmol of dibenzalacetone dissolved in 5 mL of formic acid. The reaction was heated in the presence of a condenser with continuous stirring for 24 h. The resulting solution was then cooled after the completion of the reaction and poured over crushed ice to give yellow crystals of compound **4** in an 80% yield, mp. 120–122 °C.

**Preparation of α-aminophosphonate derivatives 7a, b.**

The three-component reaction takes place between the two amines **5a** and **b** (0.01 mol), the compounds **4** (0.01 mol) and triphenylphosphite **6** (0.01 mol). The three components were dissolved in 30 mL of acetonitrile, followed by adding a few drops of perchloric acid with continuous stirring for 24 h, the completion of the reaction was followed by *TLC*. The solvent was evaporated under low pressure at a low temperature, and the remaining amount was neutralized with diethyl ether to give *α*-aminophosphonates **7a** and **b** at 90 and 92% yields.

**Diphenyl** ((**5-phenyl-3-styryl-4,5-dihydro-*1H*-pyrazol-1-yl**)(**pyridin-2-ylamino**)**methyl**)**phosphonate** (**7a**)**.**

Pale yellow powder, 90% yield, m.p. = 253–255 °C, Rf = 0.82 (5% EtOAc in CHCl_3_). ^1^H-NMR (500 MHz, DMSO-d_6_) δ: 3.08, 3.72 (2H, d, *J* = 4.5 Hz, CH_2_ of Pyrazole), 3.82, 3.85 (1H, d, *J* = 5.5 Hz, CH), 3.92–4.14 (1H, t, *J* = 3.5 Hz, CH of Pyrazole), 6.72 (1H, d, *J* = 7.2 Hz, CH), 7.54 (1H, d*, J* = 7.2 Hz, CH), 7.62- 8.11 (24H, m, Ar-H), 8.75 (1H, brs, NH); ^13^C NMR (500 MHz, CDCl_3_): δ: 41.07 (CH of Pyrazole), 55.34 (CH_2_ of pyrazole), 120.80, 135.82 (CH=CH), 106.49, 117.89, 120.34, 121.38, 127.02, 127.93, 128.50, 128.63, 130.13, 135.27, 138.40, 148.20, 150.32, 158.65 (Ar-CH), 154.88 (C=N of pyrazole); Anal. Calcd for C_35_H_31_N_4_O_3_P: C, 71.66; H, 5.33; N, 9.55. Found C, 71.71; H, 5.39; N, 9.61. Figures S1 and S2.

**Diphenyl** (((**4-chlorophenyl**)**amino**)(**5-phenyl-3-styryl-4,5-dihydro-*1H*-pyrazol-1-yl**)**methyl**)**phosphonate** (**7b**)**.**

White powder, 92% yield, m.p. = 290–292 °C, Rf = 0.78 (5% EtOAc in CHCl_3_). ^1^H-NMR (500 MHz, DMSO-d_6_) δ: 3.10, 3.65 (2H, d, *J* = 4.5 Hz, CH_2_ of pyrazole), 3.78, 3.80 (1H, d, *J* = 5.5 Hz, CH), 3.95–4.16 (1H, t, *J* = 3.5 Hz, CH of pyrazole), 6.65 (1H, d, *J* = 7.2 Hz, CH), 7.50 (1H, d, *J* = 7.2 Hz, CH), 7.56- 7.84 (24H, m, Ar-H), 8.50 (1H, brs, NH); ^13^C NMR (500 MHz, CDCl_3_): δ: 41.10 (C of pyrazole), 55.41 (CH_2_ of pyrazole), 120.81, 135.80 (CH=CH), 114.95, 120.30, 121.35, 126.09, 127.11, 127.95, 128.45, 128.67, 129.67, 130.17, 135.28, 145.76, 150.25 (Ar-CH), 154.92 (C=N of Pyrazole); Anal. Calcd for C_36_H_31_ClN_3_O_3_P: C, 69.73; H, 5.04; N, 6.78. Found C, 69.78; H, 5.09; N, 6.85. Figures S3 and S4.

**Preparation of 3-acetyl coumarine** (**10**) **[10].**

A mixture of ethyl acetoacetate (0.5 mol) and salicydehyde (0.5 mol) was prepared, then the mixture was stirred on ice to cool down, and then 10 mL of piperidine was added while stirring. The stirring process continued at zero degrees Celsius for 4 continuous hours until a yellow precipitate formed in the form of a solid mass. After that, this mass was separated by filtration and recrystallized from absolute ethanol to obtain the target compound. Yield: 80% m.p. 125–128 °C.

**Preparation of 3-acetyl-1-aminoquinolin-2**(***1H***)**-one** (**11**)

3-acetyl-1-aminoquinolin-2 (*1H*)-one (**11**) was prepared by reacting 3-acetyl coumarine (10) (0.01 mol) with excess hydrazine hydrate (10 mL) in absolute ethanol. The mixture was heated in the presence of a condenser. The reaction was followed up by *TLC*. After completion of the reaction, the solvent was evaporated to give a pale yellow powder with a yield of 80%, mp. = 277–279 °C, and Rf = 0.75 (5% EtOAc in CHCl_3_). ^1^H-NMR (500 MHz, DMSO-d_6_) δ: 2.29 (3H, s, CH_3_ of acetyl group), 3.92 (2H, brs, NH_2_), 6.82- 7.55 (4H, m, Ar-H), 8.34 (1H, brs, CH); ^13^C NMR (500 MHz, CDCl_3_): δ: 31.05 (CH_3_ of acetyl group), 128.30, 133.34 (CH=CH), 113.12, 119.11, 128.47, 129.13, 139.92 (Ar-CH), 162.31 (CO), 196.35 (CO of acetyl group; Anal. Calcd for C_11_H_10_N_2_O_2_: C, 65.34; H, 4.98; N, 13.85. Found C, 65.39; H, 5.02; N, 13.80.

**Preparation of 3-acetyl-1-**((**4-chlorobenzylidene**)**amino**)**quinolin-2**(***1H***)**-one** (**13**)**.**

Compound **11** (0.01 mol) was reacted with 4-chlorobenzaldhyde (0.01 mol) in ethanol. The reaction mixture was then heated under reflux with a few catalytic amounts of AcOH to give 3-acetyl-1-((4-chlorobenzylidene) amino) quinolin-2(*1H*)-one (**13**) with 93% yields. The reaction was followed up by *TLC*. The solvent was evaporated, purified with column chromatography, and dried. White powder, 93% yield, m.p. = 187–189 °C, Rf = 0.57 (5% EtOAc in CHCl_3_). ^1^H-NMR (500 MHz, DMSO-d_6_) δ: 2.28 (3H, s, COCH3), 7.15–7.65 (4H, Ar-H), 7.45 (2H, dd, *J* = 6.00 Hz, H-3′, H-5′), 7.80 (2H, dd, *J* = 5.5 Hz, H-2′, H-6′), 8.30 (1H, brs, CH of quinolinone), 8.38 (1H, s, CH=N); ^13^C NMR (500 MHz, CDCl_3_): δ: 30.24 (CH_3_ of COCH_3_), 119.61, 124.26, 128.30, 128.76, 128.91, 127.95, 130.07, 130.55, 131.70, 131.84, 133.45, 134.22, 136.72 (Ar-CH), 138.88 (CH=N), 160.93 (CO of quinolinone), 195.55 (CO of COCH_3_); Anal. Calcd for C_18_H_13_ClN_2_O_2_: C, 66.57; H, 4.03; N, 8.63. Found C, 66.52; H, 3.99; N, 8.58.

**General procedures for the preparation of chalcone derivatives 15a, b.**

Compound **13** (0.01 mol) reacted with aldehydes **14a**, **b** (3-formyl pyridine and 4-chloro benazaldehyde, respectively) (0.01 mol) in ethanol. Then, under reflux, a few catalytic amount of pipridine was added, yielding chalcones **15a** and **b** in 95 and 97% yields, respectively. The reaction was followed up by *TLC*. The solvent was evaporated, purified with column chromatography, and dried.

**1-**(**4-Chlorobenzylidene**)**amino**)**-3-**(**-3-**(**pyridin-3-yl**)**acryloyl**)**quinolin-2**(***1H***)**-one** (**15a**)**.**

Brown powder, 95% yield, m.p. = > 300 °C, Rf = 0.48 (5% EtOAc in CHCl_3_). ^1^H NMR (500 MHz, DMSO-d_6_) δ: 7.05 (1H, dd, *J* = 5.00 Hz, CH), 7.72 (1H, dd, *J* = 5.00 Hz, CH), 7.50 (2H, dd, *J* = 5.00 Hz, H-3′, H-5′), 7.78 (2H, dd, *J* = 5.00 Hz, H-2′, H-6′), 8.35 (1H, s, CH=N), 8.42 (1H, brs, CH of quinolinone), 7.16–8.66 (8H, Ar-H); ^13^C NMR (500 MHz, CDCl_3_): δ: 119.55, 123.32, 124.28, 128.83, 128.93, 129.67, 130.05, 130.62, 131,44, 131.71, 131.82, 132.55, 134.20, 136.56, 143.31, 148.11, 149.48 (Ar-CH), 137.22 (COCH=CH-of chalcon group),138.88 (CH=N), 152.43 (COCH=CH-of chalcone group), 160.90 (CO of quinolinone), 185.74 (CO of chalcone group); Anal. Calcd for C_24_H_16_ClN_3_O_2_: C, 66.65; H, 3.90; N, 10.15. Found C, 66.70; H, 3.96; N, 10.21. Figures S5 and S6.

**1-**(**4-Chlorobenzylidene**)**amino**)**-3-**(**-3-**(**4-chlorophenyl**)**acryloyl**)**quinolin-2**(***1H***)**-one** (**15b**)**.**

Brown powder, 97% yield, m.p. = 287–289 °C, Rf = 0.70 (5% EtOAc in CHCl_3_). ^1^H NMR (500 MHz, DMSO-d_6_) δ: 7.03 (1H, dd, *J* = 5.00 Hz, CH), 7.75 (1H, dd, *J* = 5.00 Hz, CH), 7.48 (2H, dd, *J* = 5.00 Hz, H-3′, H-5′), 7.76 (2H, dd, *J* = 5.00 Hz, H-2′, H-6′), 7.15–7.69 (8H, Ar-H), 8.37 (1H, s, CH=N), 8.40 (1H, brs, CH of quinolinone); ^13^C NMR (500 MHz, CDCl_3_): δ: 119.55, 123.32, 124.28, 128.83, 128.93, 129.67, 130.05, 130.62, 131,44, 131.71, 131.82, 132.55, 133.20, 133.60, 136.56, 143.31, 148.11, 149.48 (Ar-CH), 137.21 (COCH=CH-of chalcone group), 138.90 (CH=N), 142.50 (COCH=CH-of chalcone group), 160.90 (CO of quinolinone), 185.74 (CO of chalcone group); Anal. Calcd for C_25_H_16_Cl_2_N_2_O_2_: C, 67.13; H, 3.61; N, 6.26. Found C, 67.18; H, 3.66; N, 6.21. Figures S7 and S8.

### 2.2. Experimental Anticancer Activity

The experimental part of the study was done on 60 healthy female albino rats. The used rats were exposed to a 12-h day/night cycle with free access to food and water. This study was approved by the Ethical Committee on Animal Care of Turabah University College (project no. # TURSP/2020/71). Rats were separated into 6 groups, 10 rats each;

**Group** (**1**)**:** Negative control group.

**Group** (**2**)**:** dosed 100 mg/kg BW of *α*-aminophosphonates (**7b** compound) orally for one month.

**Group** (**3**)**:** dosed arylidine derivative of 3-acetyl-1-aminoquinolin-2(*1H*)-one (**15b** compound) (100 mg/kg BW) orally for one month.

**Group** (**4**)**:** dosed DMBA (Santa Cruz Biotechnology, Inc., Dallas, Texas, catalogue # 57–97-6) in sesame oil (50 mg/kg BW) once by gavage (Bishayee et al. 2013).

**Group** (**5**)**:** treated daily with 100 mg/kg BW of α-aminophosphonates (**7b** compound) orally for 1 month after 4 months of single dosing of DMBA in sesame oil (50 mg/kg BW).

**Group** (**6**)**:** treated daily with 100 mg/kg BW of 3-acetyl-1-aminoquinolin-2(*1H*)-one (**15b** compound) orally for 1 month after 4 months from single dosing DMBA in sesame oil (50 mg/kg BW).

Animals were weekly weighed and twice weekly palpated to monitor tumor incidence four weeks after dosing of carcinogens. The rats were slaughtered at 5 months post-dosing of DMBA after isoflurane inhalation. All tumors were fixed with 10% neutral buffered formalin. The paraffin-embedded samples were sectioned and stained with hematoxylin (H) and eosin (E) for pathological assessment. Other parts of the tissues were held at 80 °C for molecular studies.

Blood samples were collected for:

#### 2.2.1. Biochemical Evaluation of CA15-3

All rats in this work were examined for estimation of the level of CA15-3 as a sensitive marker of breast cancer by the ELISA Kit, LifeSpan BioSciences, Seattle, WA, USA, according to the manufacturer’s instructions.

#### 2.2.2. Gene Expression

##### RNA Extraction

About 100 mg of breast tissue samples were obtained from each rat, frozen in liquid nitrogen and stored in Trizol at −70 °C. Homogenization of samples was followed, then chloroform (0.3 mL) was added. Shaking and centrifugation (12,500 rpm at 4 °C) for 20 min were done. After collecting the supernatant, it has been moved to a new set of tubes with the addition of isopropanol to the samples. After shaking, the mixture was centrifuged at 12,500 rpm at 4 °C for 15 min. RNA pellets were washed with 70% ethyl alcohol, dried up, and finally dissolved in Diethylpyrocarbonate (DEPC) water. The purity and concentration of RNA were verified spectrophotometrically at 260 nm.

##### cDNA Synthesis

For obtaining cDNA, 2 μg total RNA was added to 0.5 ng of oligo dT primer and a total volume of 11 μL of sterilized DEPC water was incubated in the PCR machine (PeX 0.5 thermal Cycler) for denaturation (10 min at 65 °C). Next, 5X RT-buffer (4 μL), 10 mM dNTPs (2 μL), and Moloney Murine Leukemia Virus Reverse Transcriptase (100 U) were added, and a total volume of 20 μL was completed by DEPC water. Finally, incubation again in the PCR machine (1 h at 37 °C, then for 10 min at 90 °C) was done to inactivate the enzyme.

##### Semi-Quantitative PCR Analysis

Selected gene primers were created using the Oligo-4 computer program and manufactured by Macrogen Company (GAsa-dong, Geumcheon-gu, Korea) (Table 1). PCR was conducted in a final volume of 25 μL, consisting of cDNA (1 μL), master mix (12.5 μL) (Promega, Madison, WI), and 10 picomolar of forward and reverse primers (1 μL). The total volume was completed to 25 μL using sterile, deionized water. PCR cycle reaction was performed for 5 min at 94 °C (one cycle), then 30 cycles each consisted of denaturation for one minute at 94 °C, annealing at definite temperature for each primer, extension for one minute at 72 °C and final extension for 5 min at 72 °C. Glyceraldehyde-3-phosphate dehydrogenase (G3PDH) mRNA expression was recorded as a housekeeping gene. Electrophoresis of the resulted PCR products was performed on a 1% agarose gel stained with ethidium bromide in Tris-Borate-EDTA buffer. Finally, the PCR products were photographed under UV light.

#### 2.2.3. Histopathological Assessment and Immunohistochemical Assessment of Bcl2 Expression in Breast Tissue

Breast tissue samples were fixed in neutral buffered formalin (10%), then dehydrated in different alcohols, then cleared in xylene. Cutting of casted tissue into 5 μm sections was done. Finally, prepared slides were stained with hematoxylin and eosin (H&E). Three high power fields per slide were examined then photographed after histopathological assessment using a Canon SX620 H digital camera, 30-2, Shimomaruko 3-chome, Ohta-ku, Tokyo 146-8501, Japan using a Wolfe S9-0982 microscope. Immunohistochemical assessment of Bcl2 was fulfilled through deparaffinization, handling for 10 min with 2% H_2_O_2_, followed by antigen retrival using heating in PBS. Blocking of non-specific sites was done using 5% bovine serum albumin. Bcl2 Antibody (Catalog # sc-56015) was added to sections and incubated overnight at 4 °C. After PBS washes, biotinylated secondary antibody (1:2000 dilution, cat # sc-2040) was added. After using 3,3-diaminobezidine tetrahydrochloride, sections were counterstained using hematoxylin and the number of reacted cells was compared to the total cell number to determine the percentage of Bcl2 immunoreactive cells [11]. The significance of three different fields per rat was calculated by ANOVA using Statistical Package for the Social Sciences (SPSS) version 21.

### 2.3. Statistical Analysis

Results were presented as means ± standard error of means (SEM). Statistical analysis was accomplished by SPSS software (SPSS, IBM, Chicago, IL, USA) using analysis of variance (ANOVA) and post hoc descriptive tests with *p* < 0.05 recorded as statistically significant. Regression analysis was evaluated by the same software.

### 2.4. Computational Study

For MOPAC16 [12], density function theory (DFT) [13] was used to minimize the target compounds at the B3LYP/6-311G correlation function. To improve the chemical structure information, we performed optimization geometry.

#### 2.4.1. The Proteins’ Structure Selection

MOE 2015 “Molecular Operating Environment” [14] was used in a docking experiment to target the thymidylate synthase active site (PDB: 6w63 [15], then errors in the active site were corrected by the structure preparation tool in MOE to add hydrogens and partial charges (Amber12: EHT). That was used for energy minimization at a root mean square gradient of 0.100. MOE Site Finder was used to recognize binding sites through simulating a protein’s putative binding site using their tridimensional structure. The MOE Site Finder module’s prediction of binding sites confirmed the binding sites established by the co-crystallized ligand in the holo-investigated protein.

#### 2.4.2. MOE Stepwise Docking Method

The water with the inhibitor molecule was detached, then the MMFF94x force field was used to add charge. The triangle matcher placement method was used to optimize the 3D structure of molecules, which creates poses. London dG was used to rescore the generated pose, then refined with MMFF94x forcefield based on the free energy in kcal/mol.

## 3. Results and Discussion

### 3.1. Chemistry

**Scheme 1:**
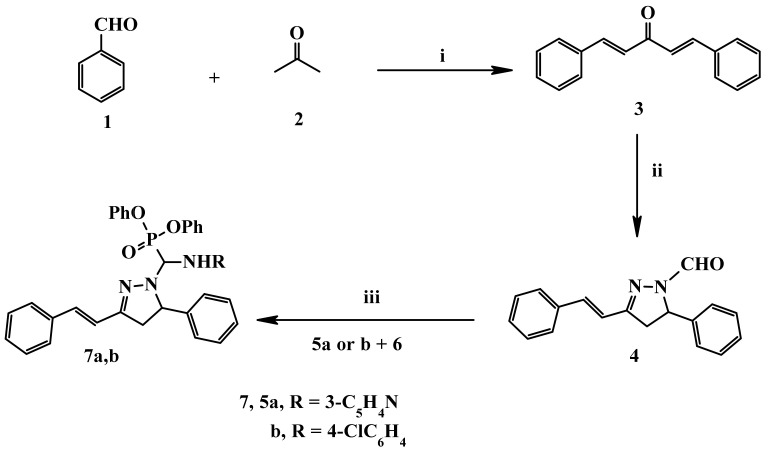
Reagent condition: (i) NaOH, EtOH, reflux, for 3: 90%. (ii) HCOOH, NH_2_NH_2_.H_2_O, reflux for 4: 80%. (iii) RNH_2_ (**5a**, **b**), (PhO)_3_P (**6**), H_3_PO_4_, CH_3_CN, reflux for **7a**: 90%; for **7b**: 92%.

Benzaldhyde **1** was reacted with acetone **2** under reflux in the presence of sodium hydroxide in absolute ethanol to give dibenzalacetone **3,** which reacted with hydrazine hydrate and formic acid under reflux to give 5-phenyl-3-styryl-4,5-dihydro-*1H*-pyrazole-1-carbaldehyde **4,** which reacted with amine derivatives **5a**, **b** and triphenyl phosphite **6** in acetonitrile with the addition of a few catalytic amounts of perchloric acid to give *α*-aminophosphonate derivatives **7a**, **b** in 90 and 92% yields, respectively (Scheme 1). The synthesized compounds **7a** and **b** were elucidated by ^1^H NMR and ^13^C NMR spectroscopic techniques. The elucidation of phosphonate derivatives **7a**, **b** by ^1^H NMR spectra showed the disappearance of CHO group and appearance of peak as douplet at 7.54 for (HN-CH-P-), peaks around 3.08–3.72 for protons of aromatic benzene ring, peaks around 3.82, 3.85 for protons of aromatic benzene ring, triplet peaks around 3.92–4.14 for methylene group of pyrazole ring, broad peak at 8.75 for NH group, and multiplet peaks around 7.62–8.11 for CH-Aromatic; ^13^C NMR spectra showed peaks around 41.07 for methene group of pyrazole ring, peak around 55.53 for (CH_2_ of pyrazole ring), peaks around 120.81 and 135.80 (CH=CH), peaks of aromatic rings appeared around 114.95 to 150.25 and peak around 154.92 for (C=N of pyrazole ring).

**Scheme 2:**
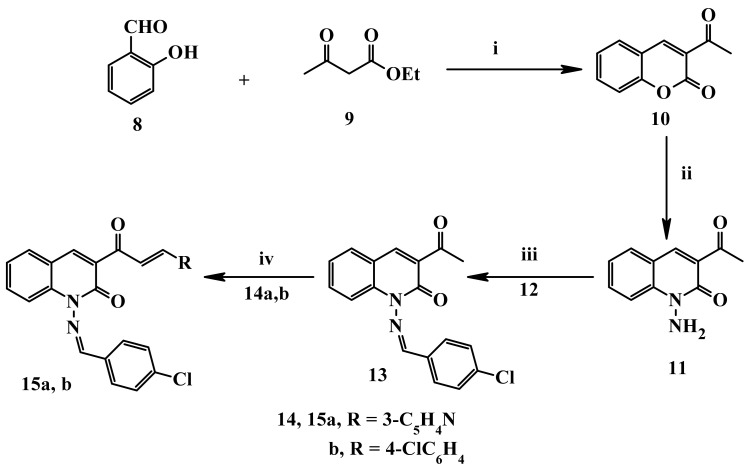
Reagent conditions: (i) Pip., EtOH, reflux, for 10: 80%. (ii) EtOH, NH_2_NH_2_. H_2_O, reflux, for 11:80%. (iii) 4-Chlorobenzaldehyde (**12**), EtOH, AcOH, reflux, for 13: 93%. (iv) RCHO (**14a**, **b**), Pip., EtOH, reflux, for **15a**: 95%; for **15b**: 97%.

Salisaldhyde **8** reacted with ethylacetoacetate **9** in absolute ethanol with the addition of a few catalytic amounts of pipridine under heating in the presence of a condenser to afford 3-acetyl coumarine **10**. In the presence of a condenser, compound **10** reacted with hydrazine hydrate in ethanol to give 3-acetyl-1-aminoquinolin-2(*1H*)-one **11** in an 80% yield. Then compound **11** reacted with 4-chloro benzaldhyde **12** in ethanol with addition of few catalytic amounts of AcOH under reflux to give 3-acetyl-1-((4-chlorobenzylidene)amino)quinolin-2(*1H*)-one **13** with 93% yields. The latter reacted with aromatic aldehydes **14a, b** in ethanol in the presence of pipridine under reflux to afford the corresponding chalcone derivatives 15a, b with 95 and 97% yields, respectively (Scheme 2) The synthesized compounds 15a and b were elucidated by ^1^H NMR and ^13^C NMR spectroscopic techniques. The elucidation of chalcone derivatives **15a, b** by ^1^H NMR spectra showed the disappearance of the acetyl group and the appearance of peaks as douplets at 7.05 and 7.72 for (CH=CH of chalcon), peaks around 8.35 for proton of (CH=N), peaks around 8.42 for (CH of quinolinone) and multiplet peaks around 7.16–8.66 for CH-aromatic; ^13^C NMR spectra showed peaks around 137.22 for (COCH=CH-of chalcon group), peaks around 152.43 for (COCH=CH-of chalcone group), peaks of aromatic rings appeared around 119.55 to 149.48, peak around 138.88 for (CH=N), appeared of peak around 160.90 for carbonyl group of chalcone and appeared of peak around 185.74 for carbonyl of quinolinone.

### 3.2. Anticancer Activity

#### 3.2.1. Results

##### Results of Gene Expression

**Figure 1:**
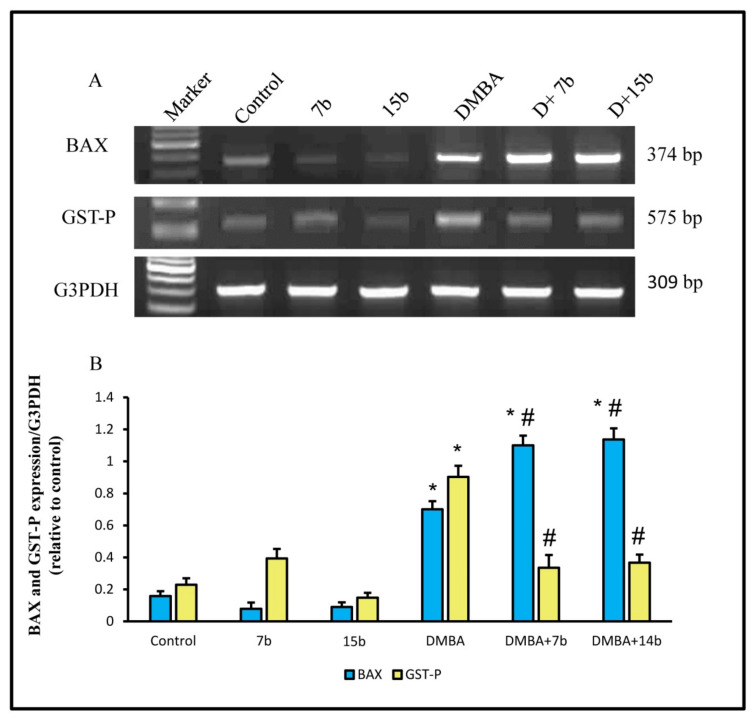
Effect of **7b** and **15b** compounds on changes in gene expression induced by DMBA in breast. Values are means ± SE of 10 rats. * *p* < 0.05 vs. control group; # *p* < 0.05 vs. DMBA group. (**A**) Upper panels are mRNA expression of examined gene. (**B**) Lower columns are densitometric analysis of gene expression.

There was no significant difference in BAX expression in the breast tissue of both negative control and DMBA-dosed female rats. Meanwhile, BAX expression in the mammary tissue of DMBA dosed female rats treated with **7b** and **15b** compounds was elevated when compared to the negative control and DMBA dosed group (*p <* 0.05) (Figure 1).

Administration of DMBA for the induction of breast cancer in adult female rats showed significant GST-P upregulation in comparison to negative control (*p* < 0.05), **7b** and **15b** compound dosed groups only. No significant difference was found between negative control, **7b**, and **15b** compound dosed groups only. Treatment of DMBA dosed female rats with **7b** and **15b** compounds resulted in normalization of mRNA expression of GST-P when compared to the DMBA dosed group (*p* < 0.05) (Figure 1).

**Figure 2:**
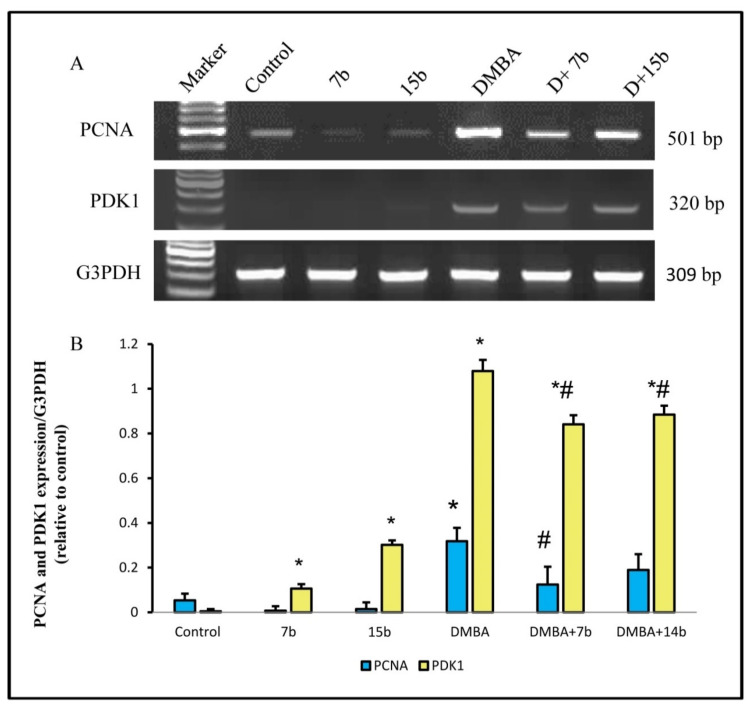
Effect of **7b** and **15b** compounds on changes in gene expression induced by DMBA in breast. Values are means ± SE of 10 rats. * *p* < 0.05 vs. control group; # *p* < 0.05 vs. DMBA group. (**A**) Upper panels are mRNA expression of examined gene. (**B**) Lower columns are densitometric analysis of gene expression.

Evaluation of the genetic effect of DMBA on PCNA mRNA expression in the breast tissue of adult female rats resulted in a significant increase in its expression in comparison to negative control *(p* < 0.05). No significant alterations were detected between the negative control, **7b**, and **15b** dosed groups only. The DMBA group treated with **7b** and **15b** showed a significant decline in the elevated mRNA expression (*p* < 0.05) (Figure 2).

The absence of PDK1 expression was observed only in the **7b** and **15b** dosed groups. DMBA administration led to a significant increase in mRNA expression of PDK1 in the breast tissue of adult female rats (*p* < 0.05). Treatment of the DMBA group with **7b** and **15b** led to a significant decline in the elevated mRNA expression in comparison to the DMBA group, particularly in the 7b group (*p* < 0.05) (Figure 2).

**Figure 3:**
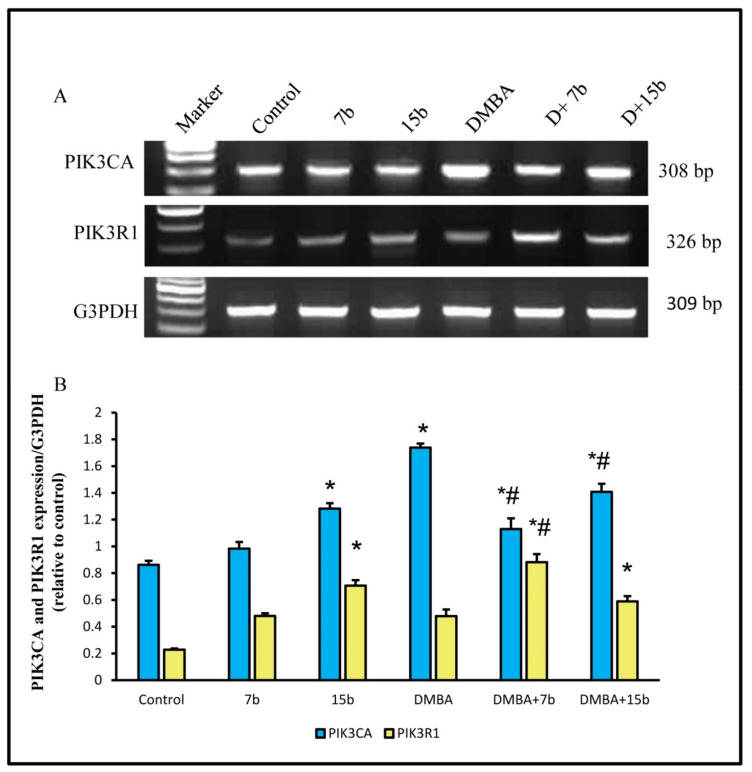
Effect of **7b** and **15b** compounds on changes in gene expression induced by DMBA in breast. Values are means ± SE of 10 rats. * *p* < 0.05 vs. control group; # *p* < 0.05 vs. DMBA group. (**A**) Upper panels are mRNA expression of examined gene. (**B**) Lower columns are densitometric analysis of gene expression.

Evaluation of PIK3CA mRNA expression in the breast tissue of DMBA administered adult female rats resulted in a significant increase (*p* < 0.05) of mRNA expression of PIK3CA in the breast tissue of adult female rats when compared to the negative control group. Treatment of the DMBA group with **7b** and **15b** led to a significant decline (*p* < 0.05) of the elevated mRNA expression when compared to the DMBA group (Figure 3).

Examination of PIK3R1 mRNA expression in breast tissue of adult female rats revealed significant elevation in **15b** administered groups mainly. However, DMBA administration showed slight elevation in comparison to the negative control group. Treatment of the DMBA group with **7b** and **15b** led to a significant elevation (*p* < 0.05) of mRNA expression of PIK3R1 when compared to the DMBA group, particularly in the **7b** group (Figure 3).

##### Histopathological Assessment

**Figure 4:**
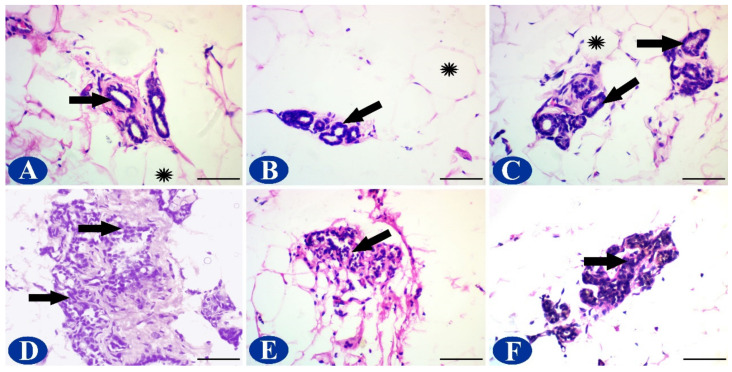
Results of histopathological assessment. (**A**) Negative control group shows the normal structure of the acini (arrow) with adipose tissue inbetween (*). (**B**) **7b** group showing normal acini (arrow) and adipocytes (*). (**C**) The **15b** group showed normal lobules (arrow) and adipose tissue (*). (**D**) Breast tissue from the DMBA group showed extensive proliferation of cancer cells (arrows). (**E**) Adult female rats administered DMBA and treated with **7b** showed marked regression of tumor mass (arrow). (**F**) breast tissue of DMBA-treated rats with **15b** revealed moderate regression of tumor mass (arrow). H & E. Scale bar = 50 μm.

Control breast tissue of negative control group showed normal acini with normal lining epithelium and adipose tissue surrounding lobules (Figure 4A). Female rats administered **7b** showed normal acinar structure with normal adipocytes (Figure 4B). The **15b** group showed the same picture as that of the negative control and **7b** groups (Figure 4C). Breast tissue from the DMBA group showed massive proliferation of cancer cells with nuclear pleomorphism and hyperchromatia (Figure 4D). Breast tissue of the DMBA and **7b** groups showed shrinkage and regression of tumor mass (Figure 4E). Breast tissue from the DMBA and **15b** groups showed moderate regression of cancer size (Figure 4F).

##### Immunohistochemical Assessment of Bcl2

**Figure 5:**
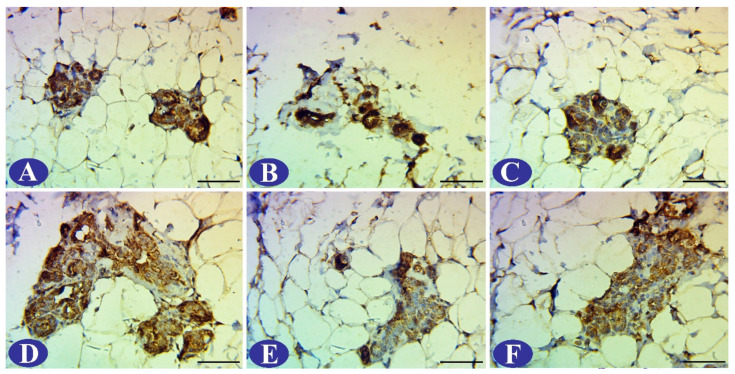
Results of Immunohistochemical Assessment of Bcl2. (**A**) Breast tissue of negative control group showed over-expression of Bcl2. The (**B**) **7b** group showed increased acinar Bcl2 expression. (**C**) **15b** group showed marked Bcl2 expression in the lobular tissue. (**D**) Breast tissue of DMBA-dosed rats revealed over-expression of Bcl2 in the tumor mass. (**E**) Breast tissue of DMBA dosed rats treated with **7b** revealed faint Bcl2 expression. (**F**) Moderate Bcl2 expression was detected in the DMBA group treated with **15b**. Scale bar = 50 μm.

In breast tissue of negative control rats, a marked expression of acinar cells with normal architecture was observed (Figure 5A). Breast tissue of the **7b** group showed a high density of Bcl2 expression in the epithelial cells of acini (Figure 5B). Breast tissue of the **15b** group showed increased Bcl2 expression in normal lobules (Figure 5C). DMBA-administered adult female rats showed marked expression of Bcl2 among the proliferated cancer cells (Figure 5D). Breast tissue of DMBA-dosed rats treated with **7b** showed regression of tumor mass with mostly faint expression of Bcl2 (Figure 5E). Finally, BMBA-administered rats treated with **15b** showed moderate regression of tumor size with moderate expression of Bcl2 (Figure 5F). The immunochemical score was documented in Table 2.

#### 3.2.2. Results of Analysis of CA 15-3

Level of CA 15-3 was significantly elevated in DMBA administered group (37.1 ± 0.3 U/mL) in comparison to negative control (14.9 ± 0.8 U/mL), **7b** group (15.1 ± 0.7 U/mL) and **15b** (16.02 ± 0.8 U/mL). Treatment with **7b** and **7b** significantly ameliorated CA 15-3 levels to 25.1 ± 0.1 and 29.8 ± 0.7 U/mL, respectively (Table 3).

### 3.3. Docking Studies

The docking study focused on thymidylate synthase to investigate the tiny compounds’ mode of action as anticancer agents. The interaction behavior between ligand and protein was detected by the MOE docking score derived from MOE 2015.10 [14]. The calculated energy is represented in Table 4. The compounds **7a**, **7b, 15a**, and **15b** fit into (PDB:6W63) the active site. Amber12: EHT force field used for energy minimization in order to obtain docked pose. The maximum MOE-scoring function evaluated the binding affinities for **7a**, **7b**, **15a**, and **15b** (Table 4). The **7a**, **7b**, **15a**, and **15b** exhibited binding affinity with TM as (−6.34, −6.2, −5.7, and 5.5 Kcal/mol), respectively (Table 4).

**Figure 6:**
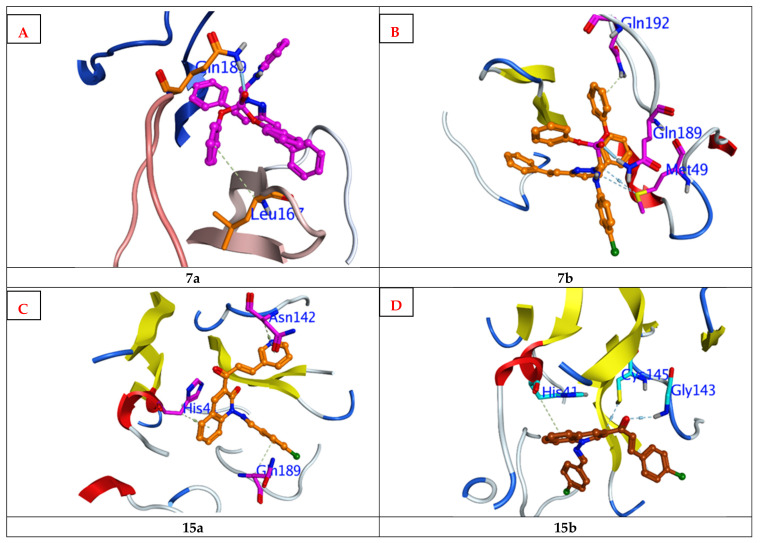
Molecular docking of the active compounds (**A**) for **7a**, (**B**) for **7b**, (**C**) for **15a**, and (**D**) for **15b** against Thimidylate synthase protein (6w63).

**Figure 7:**
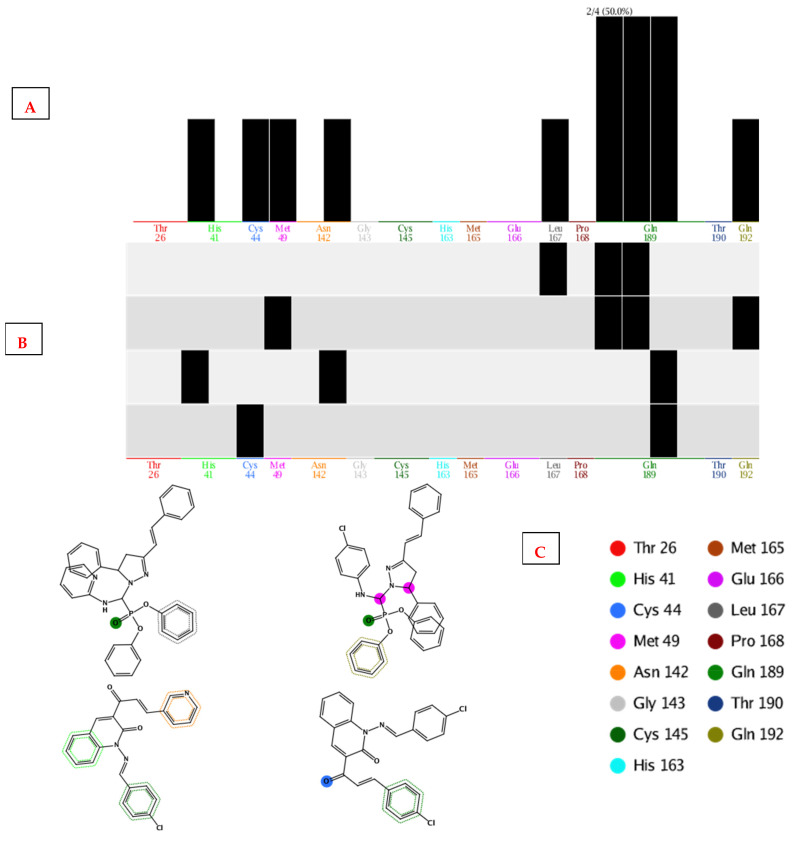
(**A**) PLIF histogram plotted, (**B**) statistical key interaction, (**C**) interaction key for (**7a**, **7b**, **15a**, and **15b**) with active site.

These compounds combined with important amino acid residue (Gln189) by formation of strong H-bonds with equal bond distances about 2.9° (Figure 6 and Figure 7). The variation in the interaction mode between **7a**, **7b**, **15a**, and **15b** and the hydrophilic amino acid backbone (Figure 6 and Figure 7) postulated that the hydrophobicity and membrane permeability play a circular role in the absorption of the molecule in biological systems.

#### In Silico Pharmacokinetic Profile

Oral bioavailability plays a vital role in the enhancement of therapeutic bioactive molecules. The ADMET (absorption, distribution, metabolism, and excretion- toxicity) Factors prevent several powerful therapeutic agents from being used in the clinic trial field. All calculated descriptors were performed using MOE and the admet-SAR package. The obtained results have been disclosed in Table 5. The ADMET properties of tested compounds were performed using calculated Lipinski rules [16], percent absorption (%ABS) [17], and topological polar surface area (TPSA), which is linked to drug bioavailability. The passively absorbed molecules with TPSA > 140 have low oral bioavailability [18]. The tested compounds showed the (6–10) values for H-bond acceptors and the H-bond donors in a range of 1 and 5. That indicated the high flexibility of these compounds. The “Clog P” lipophilicity character is lower than 5.0 [19].

These compounds fulfill Lipinski’s rule. The TPSA values (Table 5) have shown that the synthesized compounds have good drug absorption against the different parameters depicted in Table 5. The carcinogenic behavior of the synthesized compounds was investigated through comparison with 981 different carcinogenic chemical structures obtained from the “Carcinogenic Potency Database (CPDB)”. The results exhibited no carcinogenicity effect with ranged values (~None and 0.8 mg/kg body wt/day). In general, the synthesized compounds have good oral bioavailability, high BBB transport ability, and no significant health effects for rodent toxicity profiles.

## 4. Discussion

Breast cancer is the most commonly detected female cancer on a global scale, which is life-threatening to women worldwide [20]. In our study, we used DMBA (7,12-dimethylbenz(a)anthracene) as a chemical carcinogen extensively used to generate breast cancer model in rats [21,22]. We used rat model in this work because of easy detection of tumor masses by palpation and plentiful blood collection. Mammary glands using cytochrome p450 enzymes shares in the deposition and activation of DMBA through a different steps; DMBA is transformed to DMBA-3,4-diol-1,2-epoxide which reacts with DNA to create adducts that is the main reason for mutagenicity and carcinogenicity [23]. DMBA induces, oxidative stress in rats through disruption of tissue redox balance [24,25]. The resulting effect is the induction of carcinogenesis as confirmed by histopathological examination. Immunohistochemical examination of Bcl2 as antiapoptotic protein was carried out in breast tissue which showed increased expression in DMBA group. BCL2 is responsible for suppression of the mitochondrial pathway activation to apoptosis, and its expression is increased in most breast cancer cases [26]. Treatment with **7b** mainly reduced BCL2 activity, thus induces apoptosis, and limits cancer progression [26,27,28]. The reduction of cancer size may be explained by the downregulation of Bcl2 and up-regulation of BAX, which leads to a shift in the Bcl2/BAX ratio towards a pro-apoptotic signal in **7b**-treated group. Among the evaluated parameters, Bcl2 gene, and its protein got more attention as promising predictive marker. Many studies confirmed the potency of apoptosis in tumor regression and aggressiveness. The apoptotic mechanism is organized by several genes, involving activation of different proto-oncogenes, such as Bcl2 gene [29]. This gene is associated with growth and apoptosis regulation, thus plays a vital role in tumor resistance to anticancer agents, by influencing the severity of tumor cell apoptosis or by modifying cancer cells proliferation [30,31]. Bcl2 is a proto-oncogene which encodes its protein, that binds to a cell membrane, and controls cell death through apoptosis [30]. Treatment with **7b** mainly and to a lesser degree **14b**, resulted in up-regulation of Bax expression and down-regulation of Bcl-2; as vital controllers of tissue apoptosis. Therefore, leading to stimulation of the apoptotic activity in the cancerous cells [32].

Additionally, numerous studies performed on different cancer cells have determined phosphonates and coumarin induce apoptosis in the cells, and they not only decreased Bcl-2 expression but also increased Bax in cancer cells [32,33]. Tumor markers are substances found in the patient’s blood, tissue, fluid and excretions generated by cancer cells, which mainly suggest the incidence of a tumor and serve for prognosis and control of cancer cases [34]. Tumor markers are used also to identify the presence and progression of cancer [35]. Cancer Antigen 15-3 (CA15-3) is a type of transmembrane mucin glycoproteins, having variable numbers of repeats and is mostly exists in breast cancer than normal tissue [36]. Now, CA15-3 is the one of the most extensively used serum tumor marker in clinical diagnosis and early screening of human breast cancer [37]. The experimental animals showed significant difference in CA15-3 level between clinically healthy and those with mammary carcinogenesis [38]. CA 15-3 is produced by tumor cells and is used for observing advanced disease [39].

Glutathione S-transferase Pi (GST-P) was raised in DMBA administered group. GST-P has been reported to be a marker of dysplastic and preneoplastic lesions in experimental animal model of carcinogenesis. GST-P expression is modified in early and progressed cancer cases. A broad variety of invasive cancers revealed increased expression of GST-P [40]. Treatment with **7b** and **14b** after DMBA resulted in down-regulation of GST-P expression that indicates better prognosis. Proliferating cell nuclear antigen (PCNA) expression markedly increased in DMBA-administered group. PCNA is frequently used as a cell proliferation marker. Also, it has numerous functions concerning DNA replication and repair. Also it maintains genomic integrity at genetic and epigenetic levels by reacting with chaperone proteins [41,42]. Other studies showed that PCNA was up-regulated in tumor, and high PCNA levels related to poor prognosis. Some assays concluded that knockdown of PCNA repressed breast cancer cells migration and invasion [41]. Pyruvate dehydrogenase kinase 1 (PDK1) enzyme is responsible for this metabolic switch by phosphorylating pyruvate dehydrogenase [43,44]. PDK1 is over-expressed in DMBA-administered group. PDK1, is implicated in various cancers, including breast cancer [45,46]. The study of Du et al. indicated that, PDK1 is highly expressed in breast lumps in animal models and would further regulate tumor invasion and metastasis, and it is also observed that in vitro inhibition of PDK1 can suppress cancer cell growth [46] as shown in treated groups with **7b** and **14b**. Liu et al. [47] detected positive level of PDK1 protein in 213 tumor samples. Also, other studies reported that overexpression of PDK1 enhances the initiation of breast cancer [48], while suppression of PDK1 expression leads to the inhibition of breast cancer growth as seen in **7b** and **7b** group [49]. PDK1 Up-regulation leads to the incidence and progression of tumors. The phosphatidylinositol-4-5-bisphosphate-3-kinase catalytic subunit-α (PIK3CA) gene was over-expressed in DMBA-administered rats. PIK3CA encodes the p110α subunit of class IA PI3K to phosphorylate phosphatidylinositol-4,5-bisphosphate (PIP2) and transforms it into phosphoinositide 3,4,5 trisphosphate (PIP3), and subsequently stimulates further downstream pathways that finally leads to advanced cell growth, proliferation and resistance [50,51,52]. Activation of oncogenes as PIK3CA and loss of tumor suppressors are thought to be early breast cancer events. In human cancers, PIK3CA is the commonly mutated gene which encodes the p110α catalytic subunit of the PI3K pathway, and was over-expressed in breast cancer [53]. Lately, the Food and Drug Administration (FDA) approved testing of PIK3CA mutations in breast cancer patients using breast tumor tissue or circulating tumor DNA, isolated from plasma specimens [54]. Treatment of DMBA group with **7b** and **14b** resulted in significant reduction of PIK3CA expression which could be one of the factors that hinder tumor progression and facilitate its treatment. PIK3R1 expression was declined in DMBA-administered rats. PIK3R1 down-regulation was found in human breast cancer and, deletion of liver-specific Pik3r1 led to progression of PI3K pathway-activated hepatocellular carcinoma [55,56]. PIK3R1 is missed at the mRNA expression patterns in breast cancer [57]. Overexpressed PIK3R1 was detected in DMBA groups treated with **7b** and **14b** that indicated an elevated cell apoptosis and reduced cell survival in breast cancer and confirm its effect as a tumor suppressor agent [58]. Further work is required with different doses of these compounds with choice of the best dose and their evaluation on different types of animal models.

### 4.1. Docking Studies

The docking study targeted thymidylate synthase to examine the mode of action of the small compounds as antitumor agents. The ligand–protein interaction behavior was estimated based on the docking score function as implemented in MOE 2015.10 [14]. All calculations of the docking experiment are represented (Table 1). The crystal structures of Thymidylate synthase (PDB: 6w63 [15] were obtained. The compounds **7a**, **7b**, **15a**, and **15b** have been docked into the active sites of receptors. The ligands have formed complexes with the active sites of both enzymes. The extracted docked poses of ligands were energy-minimized with the molecular mechanics (Amber12: EHT) force field until the gradient convergence reached 0.05 kcal/mol. The highest MOE scoring function for tested compounds was applied to evaluate the binding affinities of tested compounds (Table 4). The compounds (**7a**, **7b**, **15a**, and **15b**) have exhibited binding affinity with TM (−6.34, −6.2, −5.7, and 5.5 Kcal/mol), respectively (Table 1).

**7a**, **7b**, **15a**, and **15b** combined with an important amino acid residue (Gln189) for the TM active site, which formed strong hydrogen bonds with equal bond distances of about 2.9° (Figure 6 and Figure 7, Table 4). These compounds were arranged in parallel mode with Glu126, where there was a hydrophilic key for stabilizing these compounds in the receptor.

The different interaction modes of ligands with hydrophilic amino acid backbones in the binding site (Figure 1, Figure 2, Figure 3, Figure 4) postulate that hydrophobicity and membrane permeability are important pharmabiotic characteristics for absorption molecules in biological systems.

#### In Silico Pharmacokinetic Profile

Oral bioavailability plays a vital role in the enhancement of therapeutic bioactive molecules. The ADMET Factors prevent several powerful therapeutic agents from being used in the clinic trial field. All calculated descriptors were performed using MOE and the admet-SAR model. The obtained results have been disclosed in Table 5. The ADMET properties of tested compounds were performed using calculated Lipinski rules [16], percent absorption (%ABS) [17], and topological polar surface area (TPSA), which is linked to drug bioavailability. The passively absorbed molecules with TPSA > 140 have low oral bioavailability [18]. The tested compounds showed the (6–10) values for H-bond acceptors and the H-bond donors in a range of between 1 and 5. That indicated the high flexibility of these compounds. The “Clog P” lipophilicity character is lower than 5.0 [19]. These compounds fulfill Lipinski’s rule. The TPSA values (Table 5) have shown that the synthesized compounds have good drug absorption against the different parameters depicted in Table 5. The carcinogenic behavior of the synthesized compounds was investigated through comparison with 981 different carcinogenic chemical structures obtained from the “Carcinogenic Potency Database (CPDB)”. The results exhibited no carcinogenicity effect with ranged values (~None and 0.8 mg/kg body wt/day). In general, the synthesized compounds have been a good oral bioavailability was high ability BBB transport, and no marked health effects observed for rodent toxicity profiles.

## 5. Conclusions

Treatment with *α*-aminophosphonates and arylidine derivatives of 3-acetyl-1-aminoquinolin-2(*1H*)-one showed a marked anticancer effect represented by a markedly regressed tumor mass with increased apoptosis mostly with *α*-aminophosphonates. The molecular docking revealed inhibition of Thymidylate synthase by these compounds.

## Figures and Tables

**Table 1:** Sequence of selected primers in this study.

Primer	Forward	Reverse
**G3PDH**	AGATCCACAACGGATACATT	TCCCTCAAGATTGTCAGCAA
**BAX**	ACCAAGCTGAGCGAGTGTC	ACAAAGATGGTCACGGTCTGCC
**GST-P**	GCTGGAGTGGAGTTTGAAGAA	GTCCTGACCACGTCAACATAG
**PCNA**	ACCTCGCTCCCCTTACAGT	GTCCCGGCATATACGTGCAA
**PDK1**	AAATGCGAAATCACCAGGAC	ATATGGGCAATCCGTAACCA
**PIK3CA**	GAATTGGGAGAACCCAGACA	TGTCTTTCAGCCACTGATGC
**PIK3R1**	CCCTCAGTGGACTTGGATGT	GCTGCTGGGAATCTGAAAAG

G3PDH; Glyceraldehyde-3-phosphate dehydrogenase, BAX; Bcl-2-associated X protein, GST-P; glutathione S-transferase P-form, PCNA; Proliferating cell nuclear antigen, PDK1; 3-phosphoinositide-dependent protein kinase 1, PIK3CA; phosphatidylinositol-4,5-bisphosphate 3-kinase, catalytic subunit alpha, PIK3R1; Phosphatidylinositol 3-kinase regulatory subunit 1.

**Table 2:** Bcl2 immunochemical score and staining intensity in tissue sections of breast tissues of treated groups.

Immunohistochemical Scoring of Bcl2	CNT	7b	14b	DMBA	DMBA + 7b	DMBA + 14b
	4	4	4	4	3	4
**Staining intensity**	+++	+++	+++	+++	+	++

Score 1 = (No expression; no positive stained cells per each of three high-power fields (HPF)/rat, at 40× magnification; Score 2 = (weak; 1–10 positive stained cells/HPF). Score 3 = (moderate; 11–20 positive stained cells/HPF). Score 4 = (strong; >20 positive stained cells/HPF). CNT; control, DMBA; 7,12-dimethylbenz(a)anthracene. + = (1–10 darkly stained cells per each of three HPF/rat); ++ = (11–20 darkly stained cells per each of three HPF/rat) +++ = (>20 darkly stained cells per each of three HPF/rat).

**Table 3:** Results of analysis of CA 15-3 in different groups. Values are means ± SE of 10 rats.

	Parameter	CA 15-3 (U/mL)
Group		
Negative control		14.9 ± 0.8
PH		15.1 ± 0.7
CO		16.02 ± 0.8
DMBA		37.1 ± 0.3 *
D + PH		25.1 ± 0.1 #
D + CO		29.8 ± 0.7 #

* *p* < 0.05 vs. negative control group; # *p* < 0.05 vs. DMBA group.

**Table 4:** Docking energy scores (kcal/mol) derived from the MOE for ligands (**7a**, **7b**, **15a** and **15b**).

mol	Δ_E_	Rmsd	E_Conf	E_Place	E._Int._	E ele
**7a**	−6.34715	1.363464	108.423	−34.696	−8.82087	−45.806
**7b**	−6.20573	1.476241	146.434	−39.0312	−8.42001	−45.7953
**15a**	−5.79989	2.56106	73.59413	−23.9119	−10.1267	−42.805
**15b**	−5.53233	3.908997	59.74263	−25.485	−9.13702	−45.6665

Where Δ_E_, E conf, and E place are the free binding energies for a given pose, conformer, and receptor. E._Int._: The ligand’s affinity for binding energy with the receptor, E ele: the electrostatic interaction via the receptor. RMSD; root-mean-square-deviation between docking pose and co-crystal ligand.

**Table 5:** Pharmacokinetic parameters for ligands 2 derived from the MOE and ADME-Tox.

Classification ADMET Predicted Profile ---		7a	7b	15a	15b
**Absorption**					
**Blood-Brain Barrier**	BBB+	0.9591	0.943	0.986	0.988
**Human Intestinal Absorption**	HIA+	1	1	0.997	1
**Caco-2 Permeability**	Caco2-	0.5337	0.539	0.509	0.512
**P-glycoprotein Substrate**	Non-substrate	0.6866	0.656	0.778	0.773
**P-glycoprotein Inhibitor**	Inhibitor	0.5	0.5	0.556	0.590
	Inhibitor	0.6809	0.668	0.543	0.588
**Renal Organic Cation Transporter**	Non-inhibitor	0.6674	0.655	0.774	0.765
**Aqueous solubility**		−3.559	−4.648	−4.317	−4.371
**Distribution**					
**Subcellular localization**	Mitochondria	0.6239	0.667	0.656	0.660
**Metabolism**					
**CYP450 2C9 Substrate**	Non-substrate	0.6135	0.593	0.725	0.702
**CYP450 2D6 Substrate**	Non-substrate	0.82	0.821	0.835	0.832
**CYP450 3A4 Substrate**	Substrate	0.5505	0.600	0.606	0.616
**CYP450 1A2 Inhibitor**	Inhibitor	0.5866	0.67	0.781	0.809
**CYP450 2C9 Inhibitor**	Inhibitor	0.5993	0.744	0.566	0.538
**CYP450 2D6 Inhibitor**	Non-inhibitor	08602	0.836	0.854	0.839
**CYP450 2C19 Inhibitor**	Inhibitor	0.6166	0.724	0.712	0.749
**CYP450 3A4 Inhibitor**	Inhibitor	0.6568	0.793	0.757	0.714
**CYP Inhibitory Promiscuity**	High CYP Inhibitory Promiscuity	0.7742	0.924	0.800	0.846
**Excretion**					
**Human Ether-a-go-go-Related Gene Inhibition**	Weak inhibitor	0.6256	0.557	0.905	0.895
**AMES Toxicity**	Non-inhibitor	0.8571	0.801	0.881	0.867
	AMES toxic	0.5235	0.541	0.616	0.583
**Carcinogens**	Non-carcinogens	0.835	0.702	0.808	0.790
**Fish Toxicity**	High FHMT	0.9906	0.999	0.987	0.990
**Tetrahymena Pyriformis Toxicity**	High TPT	0.9484	0.994	0.983	0.983
**Honey Bee Toxicity**	Low HBT	0.6835	0.703	0.857	0.851
**Biodegradation**	Not ready biodegradable	0.9943	1	1	0.997
**Acute Oral Toxicity III**		0.5863	0.576	0.650	0.649
**Carcinogenicity** (**Three-class**)	Non-required	0.4183	0.440	0.550	0.5292
**Toxicity**					
**Rat Acute Toxicity**	LD50, mol/kg	C	2.358	2.324	2.5503
**Fish Toxicity**	pLC50, mg/L	1.0580	0.898	0.772	1.0693
**Tetrahymena Pyriformis Toxicity**	pIGC50, ug/L	0.7246	1.290	1.313	0.7074
**Pharmacokinetic**					
**Lipinski’s acceptor**		7	6	5	4
**Lipinski’s donor**		1	1	0	0
**Lipinski’s druglike**		0	0	1	1
**Lipinski’s violation**		2	2	0	1
**Topological surface area**		76.05	63.16	62.63	49.74

## Data Availability

The data presented in this study are available on request from the corresponding author.

## Supplementary Materials

The following are available online. Figure S1. ^1^H NMR spectra (500 MHz, DMSO-d_6_) of diphenyl((5-phenyl-3-styryl-4,5-dihydro-1*H*-pyrazol-1-yl)(pyridin-2-ylamino)methyl)phosphonate (**7a**). Figure S2. ^13^C NMR spectra (500 MHz, CDCl_3_) of diphenyl((5-phenyl-3-styryl-4,5-dihydro-1*H*-pyrazol-1-yl)(pyridin-2-ylamino)methyl)phosphonate (**7a**). Figure S3. ^1^H NMR spectra (500 MHz, DMSO-d_6_) of diphenyl(((4-chlorophenyl)amino)(5-phenyl-3-styryl-4,5-dihydro-1*H*-pyrazol-1-yl)methyl)phosphonate (**7b**). Figure S4. ^13^C NMR spectra (500 MHz, CDCl_3_) of diphenyl(((4-chlorophenyl)amino)(5-phenyl-3-styryl-4,5-dihydro-1H-pyrazol-1-yl)methyl)phosphonate (**7b**). Figure S5. ^1^H NMR spectra (500 MHz, DMSO-d_6_) of 1-(4-chlorobenzylidene)amino)-3-(-3-(pyridin-3-yl)acryloyl)quinolin-2(1*H*)-one (**15a**). Figure S6. ^13^C NMR spectra (500 MHz, CDCl_3_) of 1-(4-chlorobenzylidene)amino)-3-(-3-(pyridin-3-yl)acryloyl)quinolin-2(1*H*)-one (**15a**). Figure S7. ^1^H NMR spectra (500 MHz, DMSO-d_6_) of 1-(4-chlorobenzylidene)amino)-3-(-3-(4-chlorophenyl)acryloyl)quinolin-2(1*H*)-one (**15b**). Figure S8. ^13^C NMR spectra (500 MHz, CDCl_3_) of 1-(4-chlorobenzylidene)amino)-3-(-3-(4-chlorophenyl)acryloyl)quinolin-2(1*H*)-one (**15b**). Figure S9. Effect of **7b** and **15b** compounds on changes in gene expression induced by DMBA in breast. Figure S10. Effect of **7b** and **15b** compounds on changes in gene expression induced by DMBA in breast. Figure S11. Effect of **7b** and **15b** compounds on changes in gene expression induced by DMBA in breast. Figure S12. Results of histopathological assessment. Figure S13. Results of Immunohistochemical Assessment of Bcl2.

## Sample Availability

Samples of the compounds are not available from the authors.
