# Supplementary material for: Investigation of the Anticancer Effect of α-Aminophosphonates and Arylidine Derivatives of 3-Acetyl-1-aminoquinolin-2(1H)-one on the DMBA Model of Breast Cancer in Albino Rats with In Silico Prediction of Their Thymidylate Synthase Inhibitory Effect
Source: Molecules. 2022 Jan 24;27(3):756. doi: 10.3390/molecules27030756 (PMC8839308; doi:10.3390/molecules27030756)
Supplement: Supplementary file 1 [file molecules-27-00756-s001.zip › molecules-1540987-supplementary.pdf]

Supplementary Material

Investigation of the Anticancer Effect of α-aminophosphonates and Arylidine Derivatives of 3-acetyl-1-aminoquinolin-2(*1H*)-one on the DMBA Model of Breast Cancer in Albino Rats with in silico Prediction of Their Thymidylate Synthase Inhibitory Effect

Mohamed A. Nassan [1], Adil Aldharani [1], Hamada H. Amer [2,*], Ahmed Elhenawy [3], Ayman A. Swelum [4,5], Omar M. Ali [2] and Yasser H. Zaki [6,7,*]

[1] Department of clinical laboratory sciences, Turabah University College, Taif University, P.O. Box 11099, Taif, 21944, Saudi Arabia
[2] Department of chemistry, Turabah University College, Taif University, P.O. Box 11099, Taif, 21944, Saudi Arabia
[3] Department of Chemistry, Faculty of Science, Al-Azhar University, Nasr City-Cairo 11884, Egypt
[4] Department of Theriogenology, Faculty of Veterinary Medicine, Zagazig University, Sharkia, 44519, Egypt
[5] Department of Animal Production, College of Food and Agriculture Sciences, King Saud University, P.O. Box 2460, Riyadh 11451, Saudi Arabia
[6] Department of Chemistry, Faculty of Science, Beni-Suef University, Beni Suef, 62514, Egypt
[7] Department of Chemistry, Faculty of Science and Humanity Studies at Al-Quwayiyah, Shaqra University, Al-Quwayiyah, Saudi Arabia
* Correspondence: h.amer@tu.edu.sa (H.H.A.); yzaki2002@yahoo.com (Y.H.Z.)

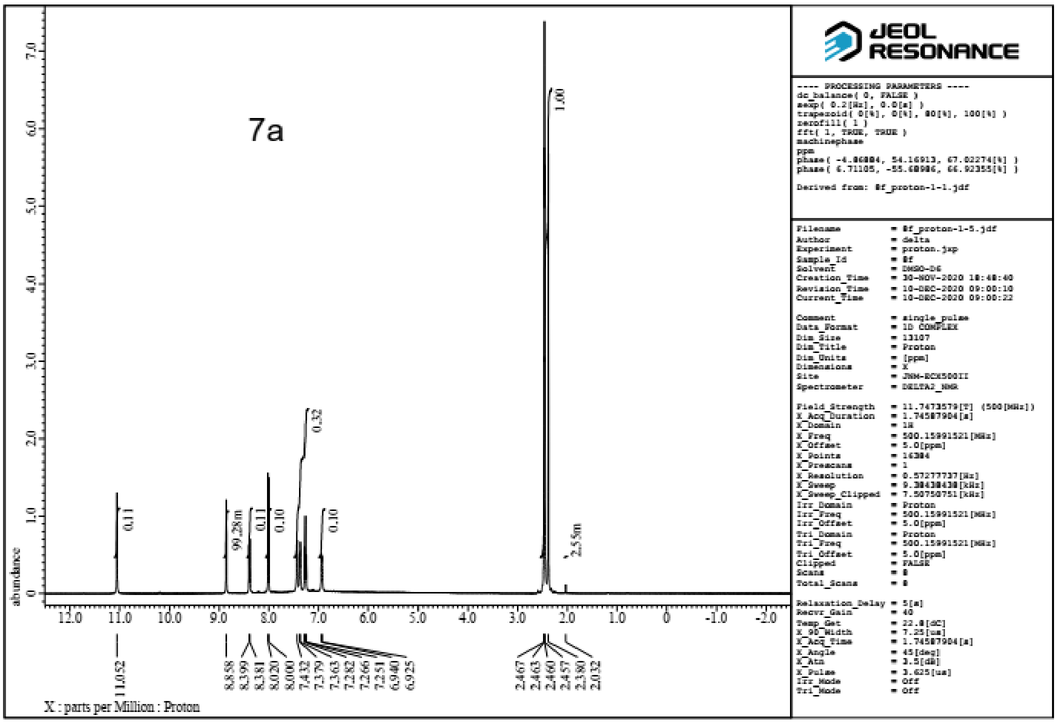

Figure S1. ^{1}H NMR spectra (500 MHz, DMSO-d$_6$) of *diphenyl ((5-phenyl-3-styryl-4,5-dihydro-1H-pyrazol-1-yl)(pyridin-2-ylamino)methyl)phosphonate* (**7a**).

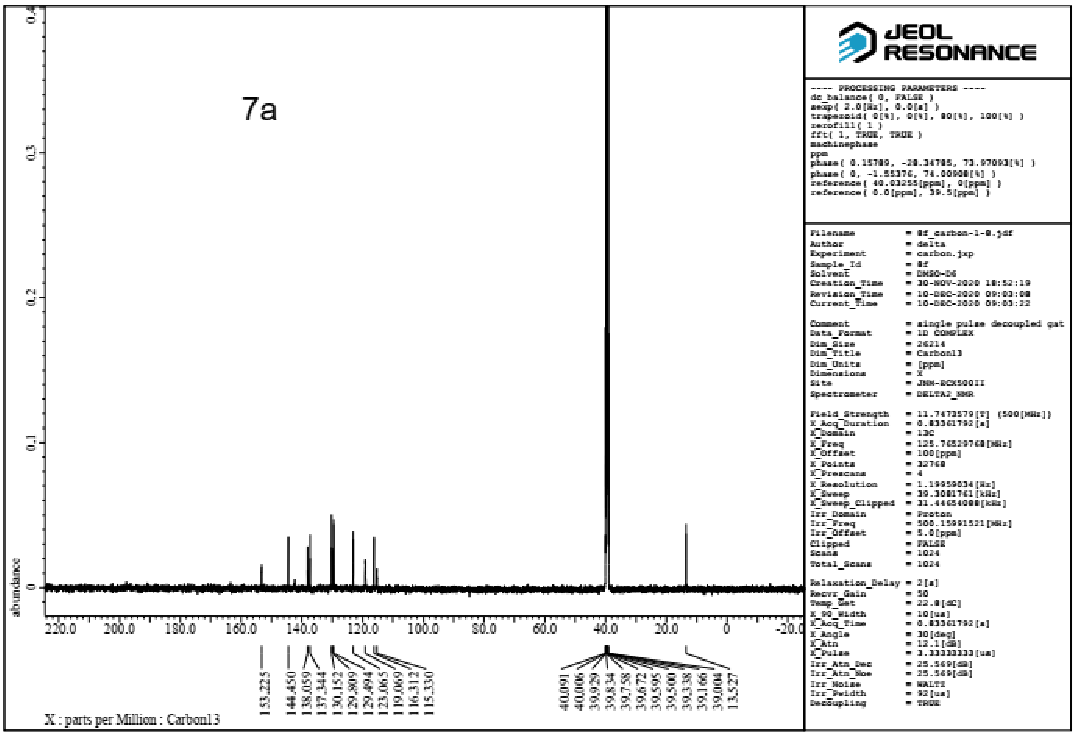

Figure S2. ^{13}C NMR spectra (500 MHz, CDCl$_3$) of *diphenyl ((5-phenyl-3-styryl-4,5-dihydro-1H-pyrazol-1-yl)(pyridin-2-ylamino)methyl)phosphonate* (**7a**).

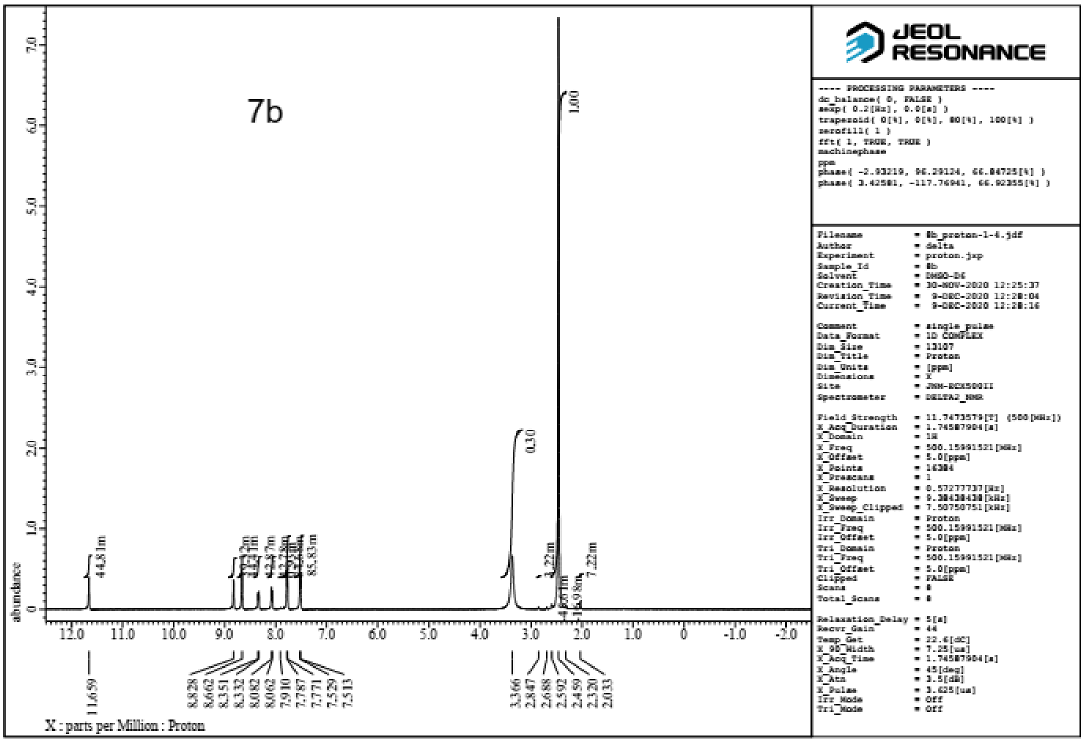

Figure S3. ^{1}H NMR spectra (500 MHz, DMSO-d_6) of *diphenyl (((4-chlorophenyl)amino)(5-phenyl-3-styryl-4,5-dihydro-1H-pyrazol-1-yl)methyl)phosphonate (**7b**).*

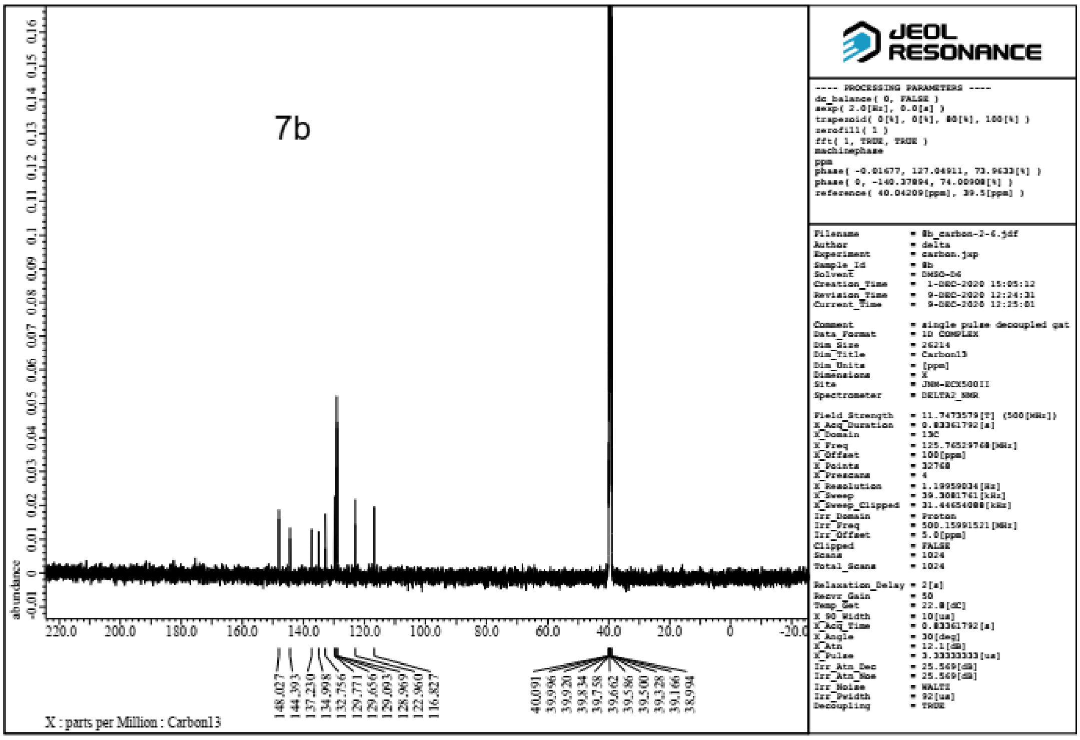

Figure S4. ^{13}C NMR spectra (500 MHz, $CDCl_3$) of *diphenyl (((4-chlorophenyl)amino)(5-phenyl-3-styryl-4,5-dihydro-1H-pyrazol-1-yl)methyl)phosphonate* (**7b**).

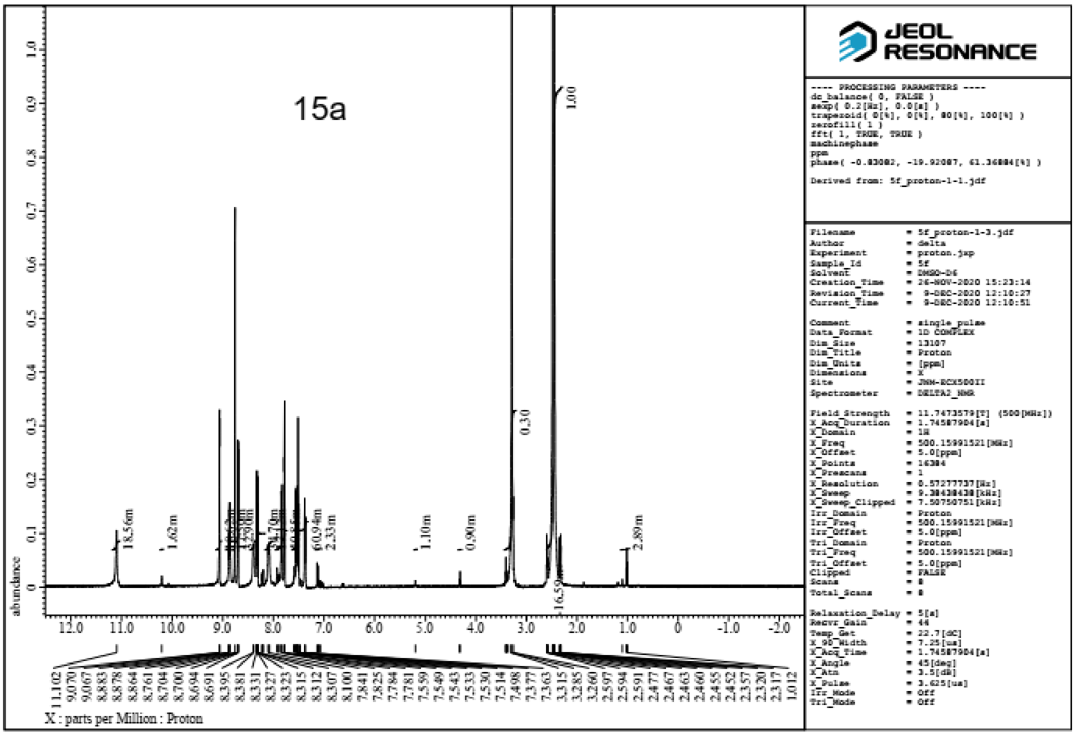

Figure S5. ^{1}H NMR spectra (500 MHz, DMSO-d_6) of *1-(4-chlorobenzylidene)amino)-3-(-3-(pyridin-3-yl)acryloyl)quinolin-2(1H)-one* (**15a**).

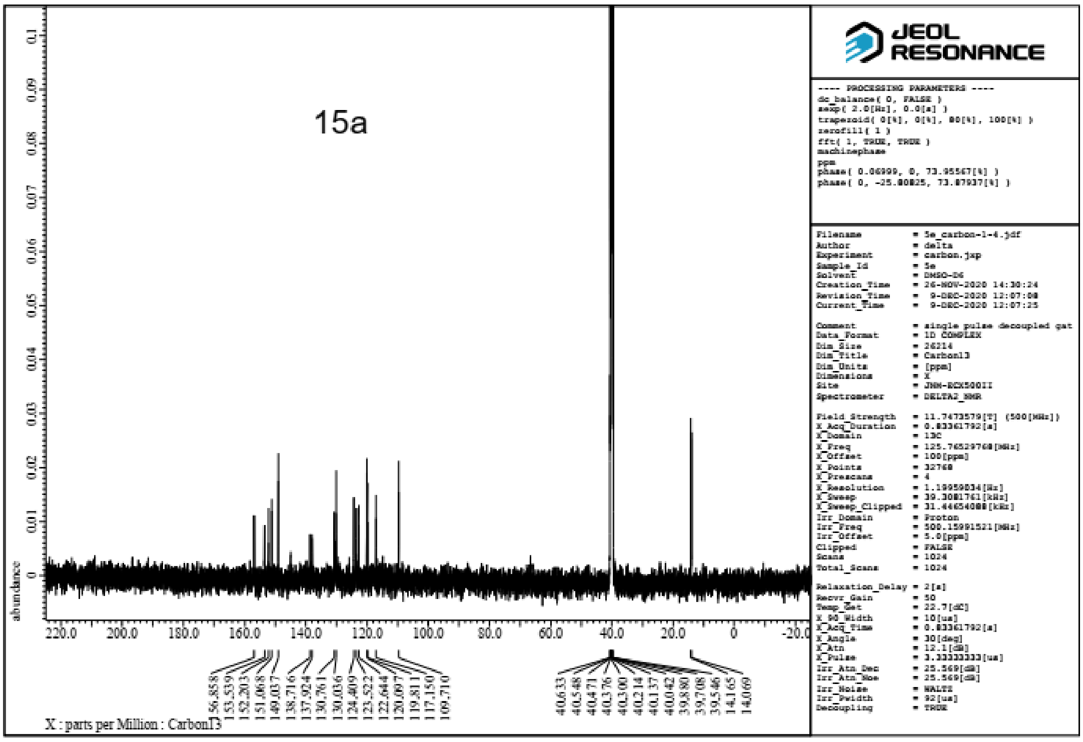

Figure S6. ^{13}C NMR spectra (500 MHz, $CDCl_3$) of *1-(4-chlorobenzylidene)amino)-3-(-3-(pyridin-3-yl)acryloyl)quinolin-2(1H)-one* (**15a**).

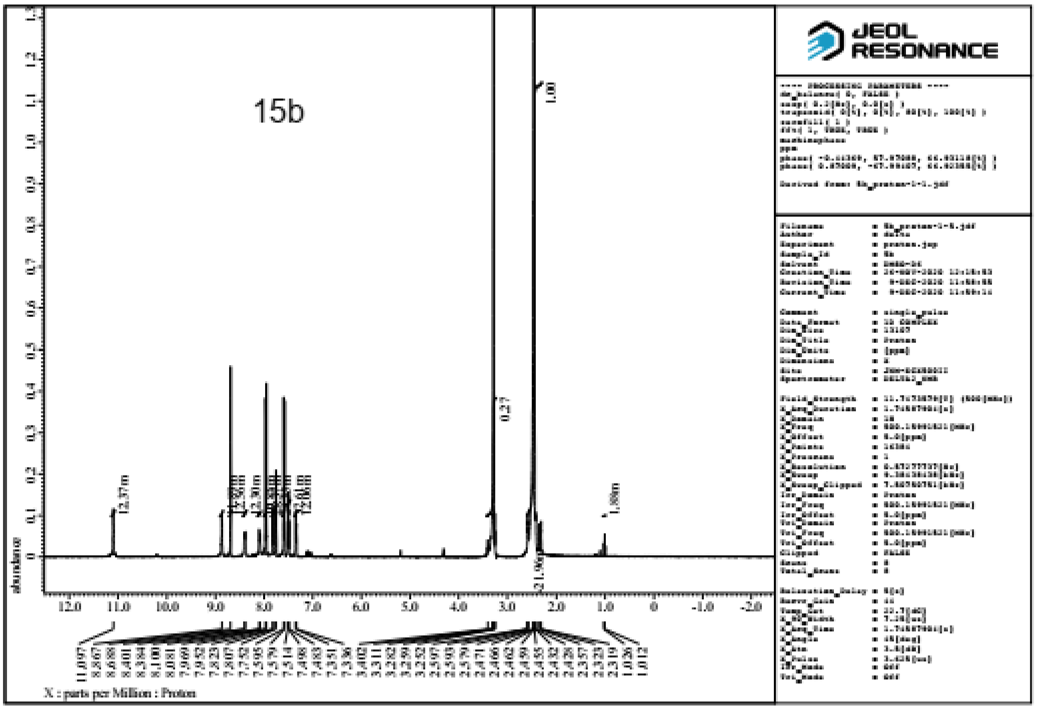

Figure S7. ^{1}H NMR spectra (500 MHz, DMSO-d_6) of *1-(4-chlorobenzylidene)amino)-3-(-3-(4-chlorophenyl)acryloyl)quinolin-2(1H)-one* (**15b**).

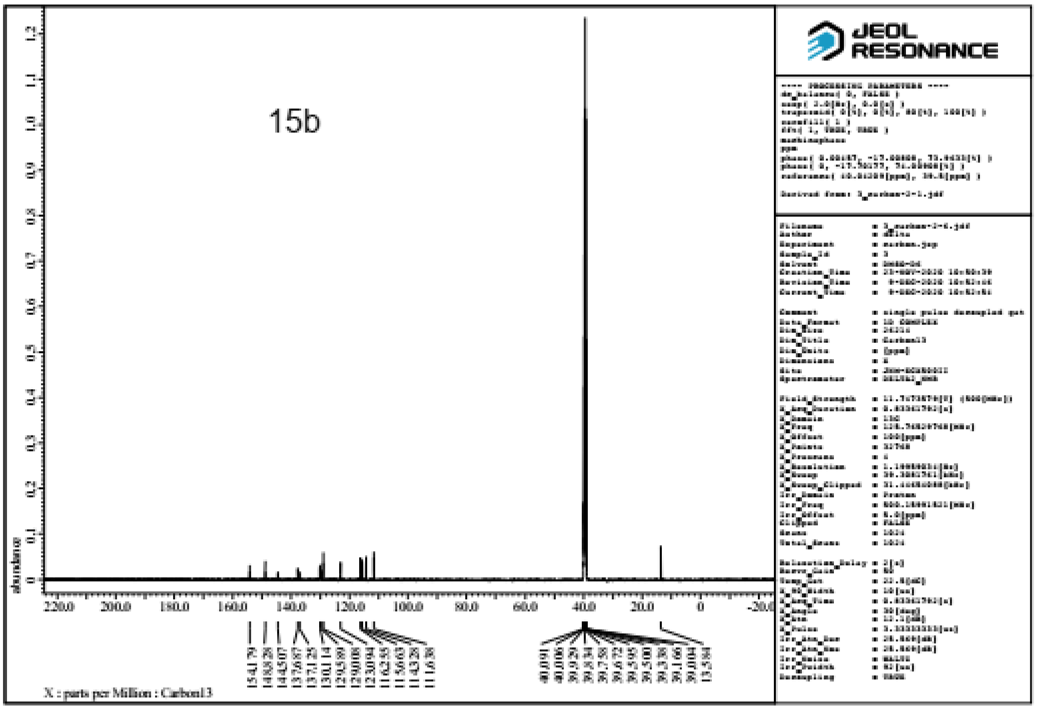

Figure S8. ^{13}C NMR spectra (500 MHz, $CDCl_3$) of *1-(4-chlorobenzylidene)amino)-3-(-3-(4-chlorophenyl)acryloyl)quinolin-2(1H)-one* (**15b**).

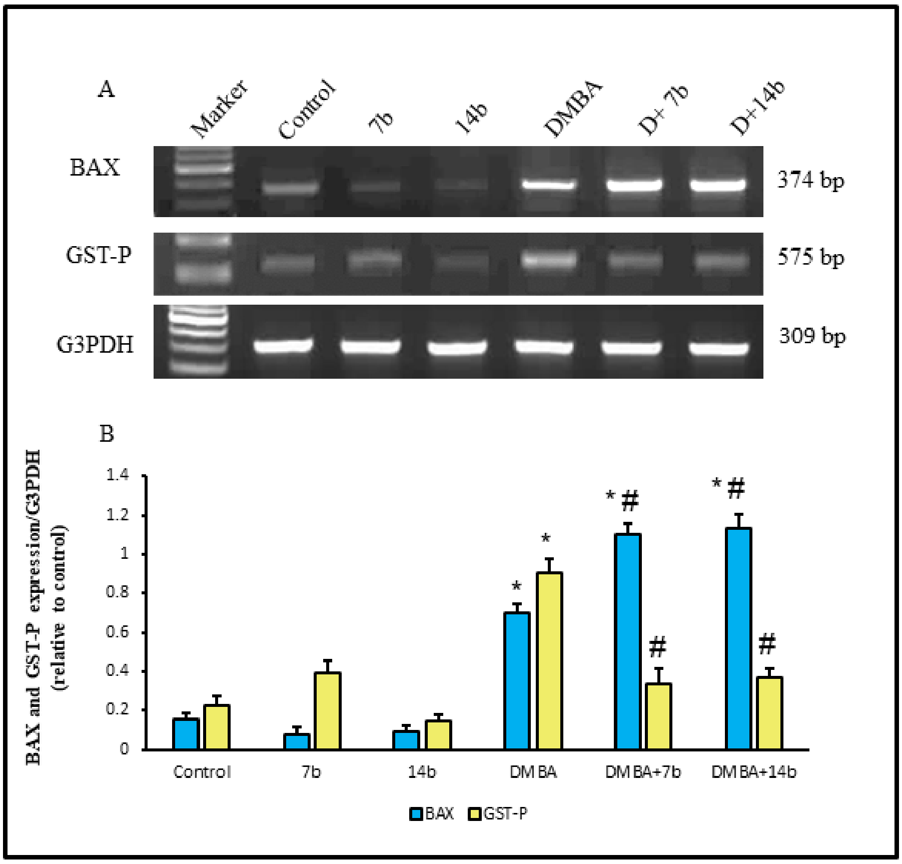

Figure S9. Effect of **7b** and **15b** compounds on changes in gene expression induced by DMBA in breast. Values are means ± SE of 10 rats. *P < 0.05 vs control group; # P < 0.05 vs DMBA group. Upper panels are mRNA expression of examined gene. Lower columns are densitometric analysis of gene expression.

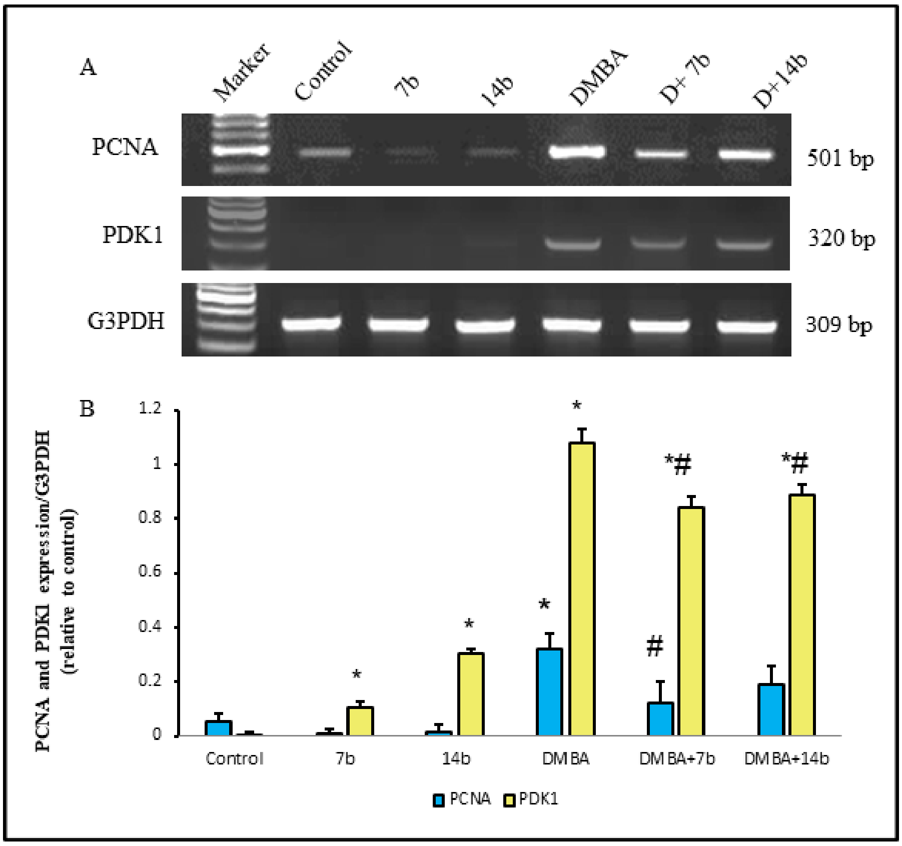

Figure S10. Effect of **7b** and **15b** compounds on changes in gene expression induced by DMBA in breast. Values are means ± SE of 10 rats. *P < 0.05 vs control group; # P < 0.05 vs DMBA group. Upper panels are mRNA expression of examined gene. Lower columns are densitometric analysis of gene expression.

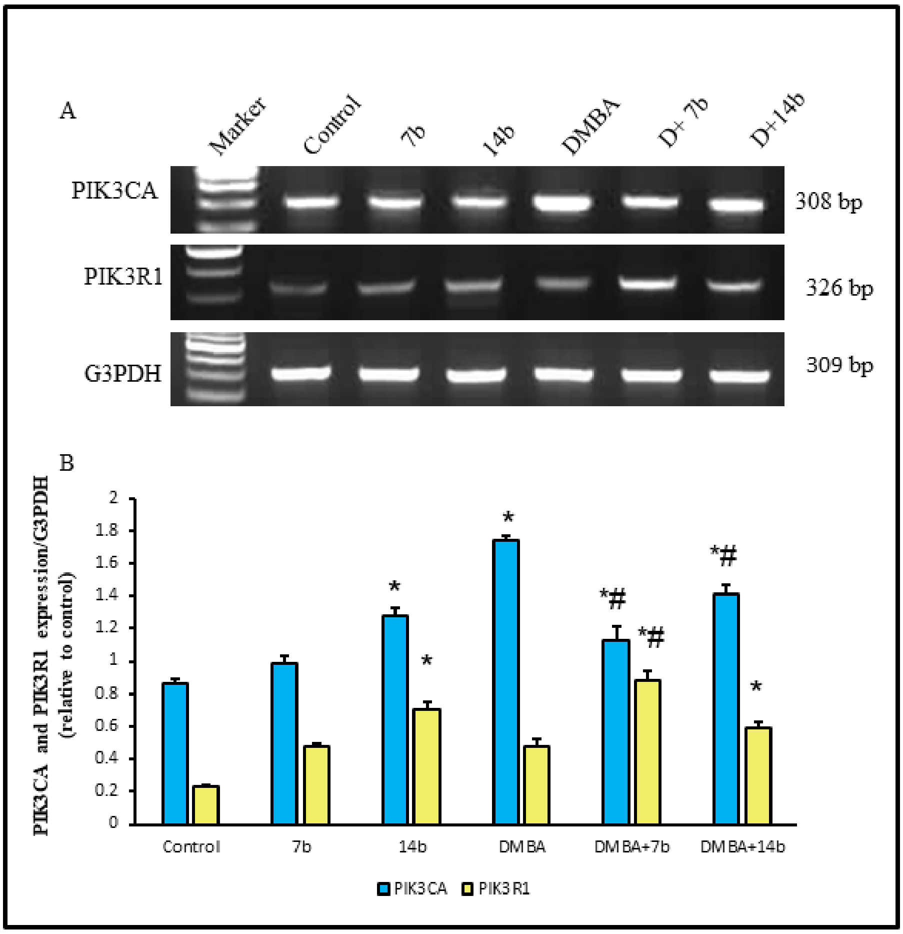

Figure S11. Effect of **7b** and **15b** compounds on changes in gene expression induced by DMBA in breast. Values are means ± SE of 10 rats. *P < 0.05 vs control group; # P < 0.05 vs DMBA group. Upper panels are mRNA expression of examined gene. Lower columns are densitometric analysis of gene expression.

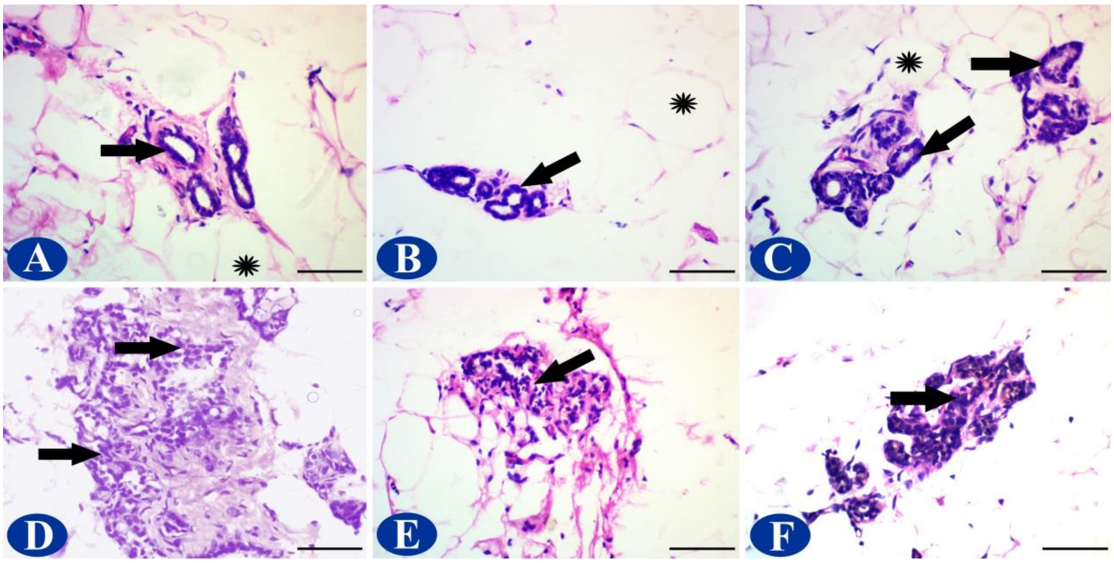

Figure S12. Results of histopathological assessment. Negative control group shows the normal structure of the acini (arrow) with adipose tissue inbetween (*). B.**7b** group showing normal acini (arrow) and adipocytes (*). C. The **15b** group showed normal lobules (arrow) and adipose tissue (*). D. Breast tissue from the DMBA group showed extensive proliferation of cancer cells (arrows). E. Adult female rats administered DMBA and treated with **7b** showed marked regression of tumor mass (arrow). F. breast tissue of DMBA-treated rats with **15b** revealed moderate regression of tumor mass (arrow). H & E. Scale bar = 50 μm.

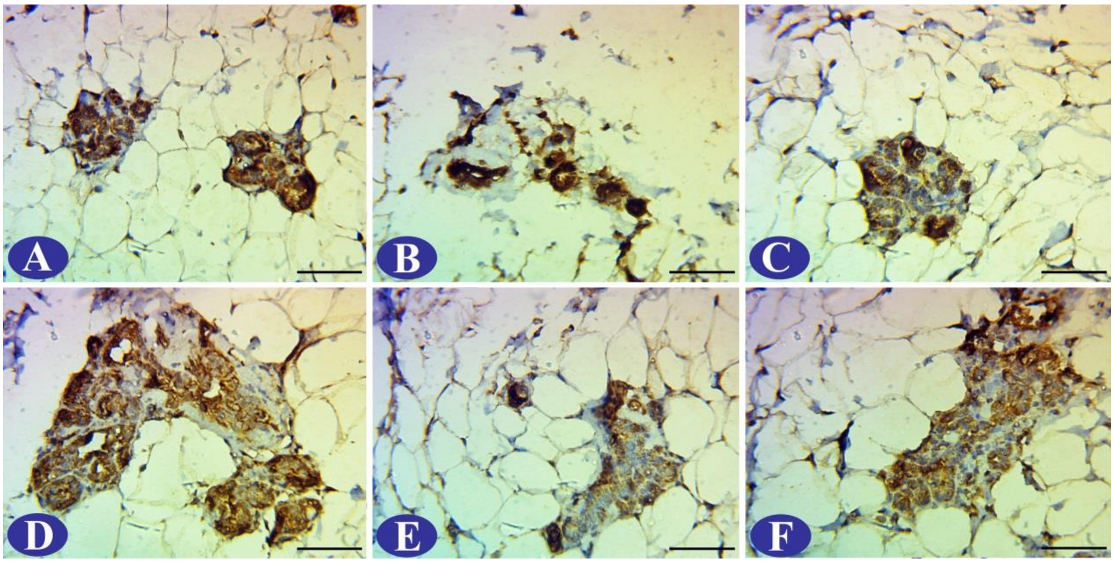

Figure S13. Results of Immunohistochemical Assessment of Bcl2. Breast tissue of negative control group showed over-expression of Bcl2. The B.**7b** group showed increased acinar Bcl2 expression. C. **15b** group showed marked Bcl2 expression in the lobular tissue. D. Breast tissue of DMBA-dosed rats revealed over-expression of Bcl2 in the tumor mass. E. Breast tissue of DMBA dosed rats treated with **7b** revealed faint Bcl2 expression. F. Moderate Bcl2 expression was detected in the DMBA group treated with **15b**. Scale bar=50 μm.
